# Synthesis and Antiangiogenic Activity of N-Alkylated Levamisole Derivatives

**DOI:** 10.1371/journal.pone.0045405

**Published:** 2012-09-14

**Authors:** Anders N. Hansen, Christine D. Bendiksen, Lene Sylvest, Tina Friis, Dan Staerk, Flemming Steen Jørgensen, Christian A. Olsen, Gunnar Houen

**Affiliations:** 1 Department of Medicinal Chemistry, Faculty of Pharmaceutical Sciences, University of Copenhagen, Copenhagen, Denmark; 2 Department of Clinical Biochemistry and Immunology, Statens Serum Institut, Copenhagen, Denmark; University of Sydney, Australia

## Abstract

Inhibition of angiogenesis is a promising addition to current cancer treatment strategies. Neutralization of vascular endothelial growth factor by monoclonal antibodies is clinically effective but may cause side effects due to thrombosis. Low molecular weight angiogenesis inhibitors are currently less effective than antibody treatment and are also associated with serious side effects. The discovery of new chemotypes with efficient antiangiogenic activity is therefore of pertinent interest. (*S*)-Levamisole hydrochloride, an anthelminthic drug approved for human use and with a known clinical profile, was recently shown to be an inhibitor of angiogenesis *in vitro* and exhibited tumor growth inhibition in mice. Here we describe the synthesis and *in vitro* evaluation of a series of N-alkylated analogues of levamisole with the aim of characterizing structure–activity relationships with regard to inhibition of angiogenesis. *N*-Methyllevamisole and *p*-bromolevamisole proved more effective than the parent compound, (*S*)-levamisole hydrochloride, with respect to inhibition of angiogenesis and induction of undifferentiated cluster morphology in human umbilical vein endothelial cells grown in co-culture with normal human dermal fibroblasts. Interestingly, the cluster morphology caused by *N*-methyllevamisole was different than the clusters observed for levamisole, and a third “cord-like” morphology resembling that of the known drug suramin was observed for an aniline-containing derivative. New chemotypes exhibiting antiangiogenic effects *in vitro* are thus described, and further investigation of their underlying mechanism of action is warranted.

## Introduction

Angiogenesis, the expansion of the blood vascular system in response to oxygen consumption and deficiency, is essential to the growth of cells and tissues. Normal physiological angiogenesis takes place during growth, wound healing, the menstrual cycle, and pregnancy [1]–[4]. Aberrant angiogenesis has been shown to play an important part of the pathological processes in cancer and other diseases such as endometriosis and rheumatoid arthritis [1], [5]–[8]. Since the idea that inhibition of angiogenesis could have therapeutic potential in relation to cancer was first suggested about 40 years ago [9], [10], it has been demonstrated to be beneficial with respect to several types of cancer and may also have therapeutic potential in other diseases associated with increased angiogenesis [5]–[8], [11]–[14]. Moreover, blood vessel normalization through antiangiogenic treatment has emerged as a possible complementary mechanism in cancer therapy [15], [16].

Vascular endothelial growth factor (VEGF), which exists in several variants and signals through a family of VEGF receptors, is the most important extracellular signalling molecule in the stimulation of blood and lymph angiogenesis [3], [17]–[19]. Currently, the most efficient inhibitor of angiogenesis in the clinic is bevacizumab (Avastin**®**; Genentech/Roche), an antibody that binds to and thereby neutralizes the effects of VEGF, which has shown beneficial clinical survival effects in several types of cancer [7], [20], [21]. Avastin treatment, however, is accompanied by an increased risk of venous thromboembolism [22] and the treatment regime is expensive. This has lead to an interest in the development of peptide-based [23] and low molecular weight angiogenesis inhibitors. Small molecules may be desirable in many respects, including improved pharmacokinetics and half-life in the human body, a decreased risk of immune response, and significantly lower production costs. Several low molecular weight angiogenesis inhibitors have been synthesized and investigated both *in vitro* and *in vivo*, as well as in clinical trials, and so far three tyrosine kinase inhibitors have gained approval by the FDA for cancer treatment [*i.e*., sorafenib (Nexavar**®**; Bayer), sunitinib (Sutent**®**; Pfizer), and pazopanib (Votrient**®**; GlaxoSmithKline] [15].

Moreover, a number of known drugs or clinical candidates with a wide variety of phenotypes have been found to inhibit angiogenesis. Examples include the fumagillin analogue TNP-470 [24], thalidomide [25], [26], nonsteroidal anti-inflammatory drugs (NSAIDs) [27], and the antifungal compound itraconazole [28].

Likewise the anthelminthic drug (*S*)-levamisole hydrochloride (Ergamisol**®**; **1**) [29], [30], which has also been used in the treatment of rheumatoid arthritis [31], [32], as well as an immunostimulant adjuvant in chemotherapy for several types of cancer [33]–[36], was recently shown to exhibit angiogenesis inhibitory activity *in vitro* and tumor growth inhibition *in vivo* [37], [38]. The *in vitro* antiangiogenic effect resembled that of Avastin in several respects, but especially with regard to inhibition of network formation and induction of non-differentiated clusters of cells [38]. In addition, levamisole is an alkaline phosphatase inhibitor [39], [40], and recent structure–activity relationship studies with synthetic analogues have addressed this capacity [41], [42]. Levamisole treatment, however, has been associated with side effects [43], and the drug was discontinued for human use in the USA in 2000, due to more efficient alternatives. In light of the recent discovery that levamisole exhibited antiangiogenic efficacy and that significant tumor growth inhibition was observed at 12 mg/kg in nude mice, we were encouraged to perform a structure–activity relationship study based on levamisole as the parent compound.

Herein, various derivatives of levamisole, obtained either through chemical synthesis or commercial sources, were tested in an *in vitro* angiogenesis assay [44] in order to identify novel lead structures and gain structure–activity relationships related to this scaffold. The cationic analogue, *N*-methyllevamisole, proved particularly efficacious with respect to induction of cluster morphology and network disruption, and thus constitute an interesting new chemotype for further investigation.

## Results and Discussion

**Figure 1:**
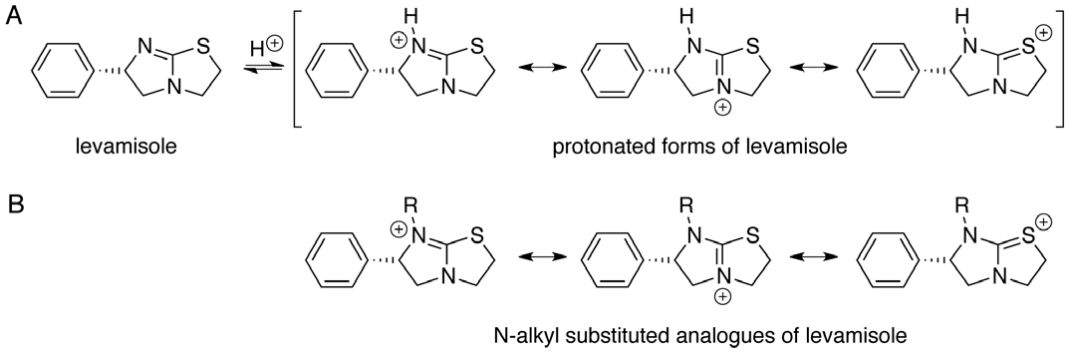
Generic structures of compounds studied. *A)* Levamisole and resonance forms of protonated levamisole; *B)* resonance forms of N-substituted analogues of levamisole.

**Figure 2:**
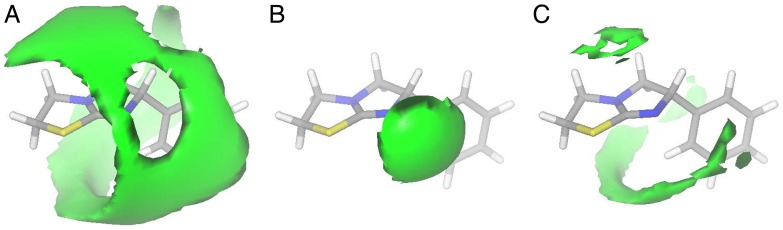
GRID calculated Molecular Interaction Fields (MIFs) for levamisole. The depicted conformation corresponds to the global energy minimum conformation of levamisole. *A)* Methyl probe, contour level –1 kcal/mol; *B)* amide nitrogen probe, contour level –5 kcal/mol; *C)* carbonyl oxygen probe, contour level –1 kcal/mol.

**Figure 3:**
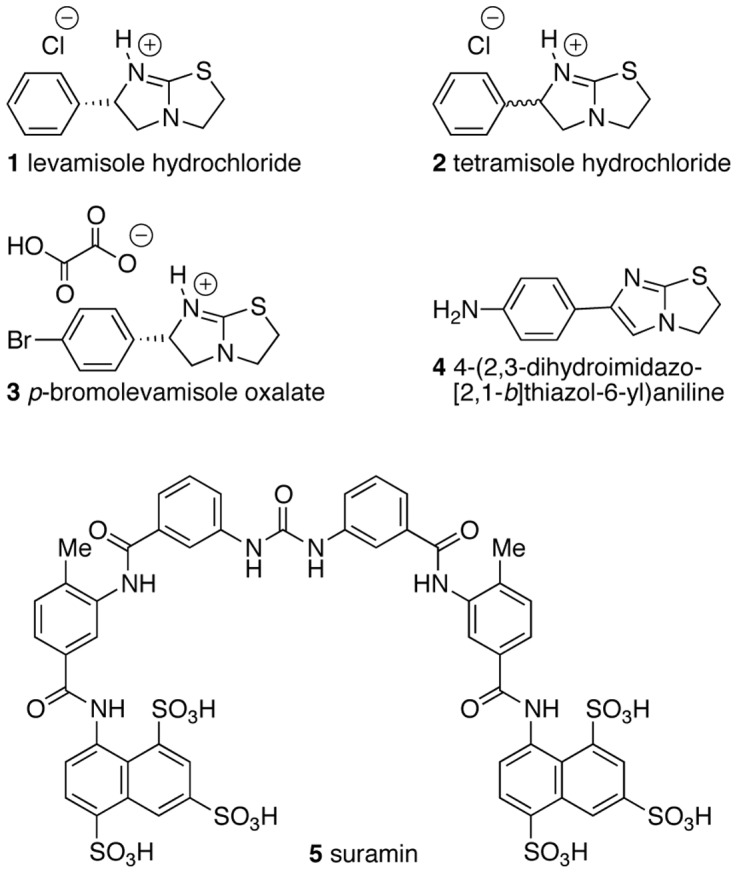
Commercially obtained compounds that were tested in this study.

Levamisole contains a benzene ring and a hetero-substituted bicycle [3.3.0] octene system (Figure 1A). Although the latter is not an aromatic system, the presence of the thiourea moiety provides the ring system with some conjugation and thereby delocalization of the carbon–nitrogen double bond as well as the lone pairs at the bridgehead nitrogen and the sulphur atoms. A conformational analysis of levamisole (molecular mechanics, MMFF96s force field) showed that levamisole preferred an “L-shaped” conformation with the two ring systems being nearly perpendicular to each other (Figure 2A–C). Due to the relatively rigid nature of this molecule, we argue that such a conformational search reflects the preferred conformation rather well. Generation and subsequent inspection of GRID calculated Molecular Interaction Fields (MIFs) [45] clearly showed that levamisole is a hydrophobic compound with only a single directional possibility for an intermolecular interaction, *i.e*., by hydrogen bonding *via* the non-bridgehead nitrogen lone pair (Figure 2B). The predicted p*K*_a_ value of levamisole was 7.0 (see, www.chemaxon.com), which is close to physiological pH and therefore indicates that both the neutral and protonated forms of levamisole are likely to be present, and should be considered equally in a structure–activity analysis. We envisioned that if, in fact, the protonated state of levamisole was responsible for its antiangiogenic effect, permanently cationic analogues obtained through N-alkylation could have potential as novel inhibitors (Figure 1B). The synthesized analogues were tested alongside a selection of commercially available compounds (Figure 3).

**Figure 4:**
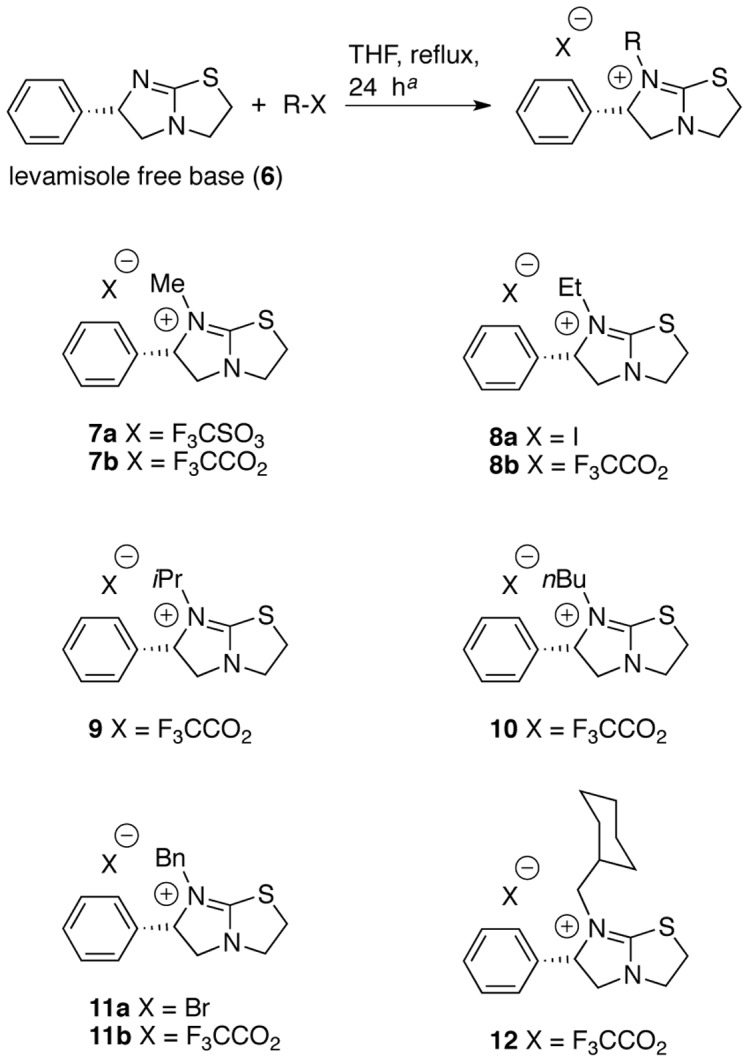
Structures of the N-alkylated analogues of levamisole synthesized and tested in this study. *^a^*Standard conditions applied for all compounds except the *N*-methylated analogue **7a**, which was furnished by treatment with methyltriflate in diethylether for 2 h at room temperature as previously described [50].

The chemistry of levamisole has been investigated to some extent, *e.g.,* it has been applied as a catalyst for enantioselective transformations [46], and N-alkylated analogues have been prepared and undergone treatment with various nucleophiles [47]–[49] or investigated as a ligand in palladium-(II) complexes [50]. Thus, in addition to the known N-alkylated analogues **7a** (methyl) [48], [50] and **11a** (benzyl) [48], we decided to vary the bulk of the alkyl group, and to investigate the effect of different counter ions (**7a–12**, Figure 4). Levamisole hydrochloride (**1**), its racemic mixture tetramisole (**2**) [(±)-levamisole], *p*-bromolevamisole (**3**), and the aromatic compound **4** are commercially available and were tested to complement the array of derivatives obtained through chemical synthesis (Figure 3). Suramin (**5**), a well-documented angiogenesis inhibitor, was included in the study as well. The preparation of the N-substituted levamisole analogues was accomplished by straightforward alkylation reactions (Figure 4) [48], [50], and the identities and purities of the synthesized compounds were confirmed by HPLC and NMR spectroscopy, respectively. Yields of the synthesized compounds were in the range of 9% (*N*-(*n*-butyl)-levamisole (**9**)) to 57% (*N*-methyllevamisole (**7**)) for the isolated compounds with purities of >95% after purification by preparative-scale reversed-phase HPLC. The synthetic yields were not optimized, as the main focus of this study was the biological evaluation of these compounds.

**Figure 5:**
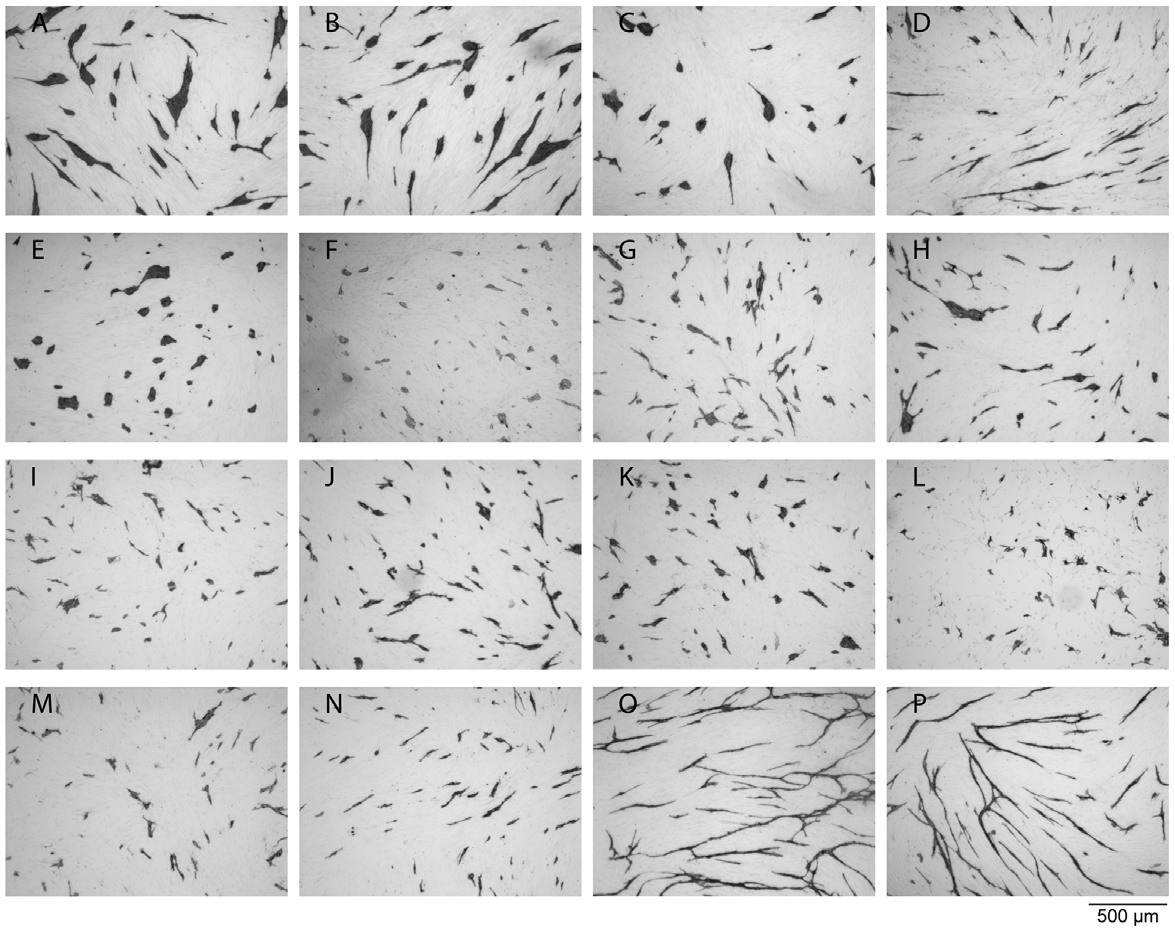
The effect of levamisole and its analogues recorded in the in vitro angiogenesis assay performed with HUVECs growing on a fibroblast monolayer. The images show HUVECs visualized by immunostaining for CD31 after treatment with: *A)* 1 mM levamisole (**1**); *B)* 1 mM tetramisole (**2**); *C)* 1 mM *p*-bromolevamisole (**3**); *D)* 1 mM compound 4; *E)* 1 mM *N*-methyllevamisole triflate (**7a**); *F)* 0.7 mM *N*-methyllevamisole trifluoroacetate (**7b**); *G)* 0.2 mM *N*-ethyllevamisole trifluoroacetate (**8b**); *H)* 0.7 mM *N*-ethyllevamisole iodide (**8a**); *I)* 0.6 mM *N*-isopropyllevamisole trifluoracetate (**9**); *J)* 0.3 mM *N*-butyllevamisole trifluoroacetate (**10**); *K)* 0.4 mM *N*-benzyllevamisole trifluoroacetate (**11b**); *L)* 0.7 mM *N*-benzyllevamisole bromide (**11a**); *M)* 0.1 mM *N*-cyclohexylmethylenelevamisole trifluoroacetate (**12**); *N)* 1 mM suramin (**5**); *O)* medium (control); *P)* 0.1 % DMSO (the control was diluted 1∶1000 corresponding the concentration of DMSO present when testing 1 mM of a compound diluted from a DMSO stock solution).

**Figure 6:**
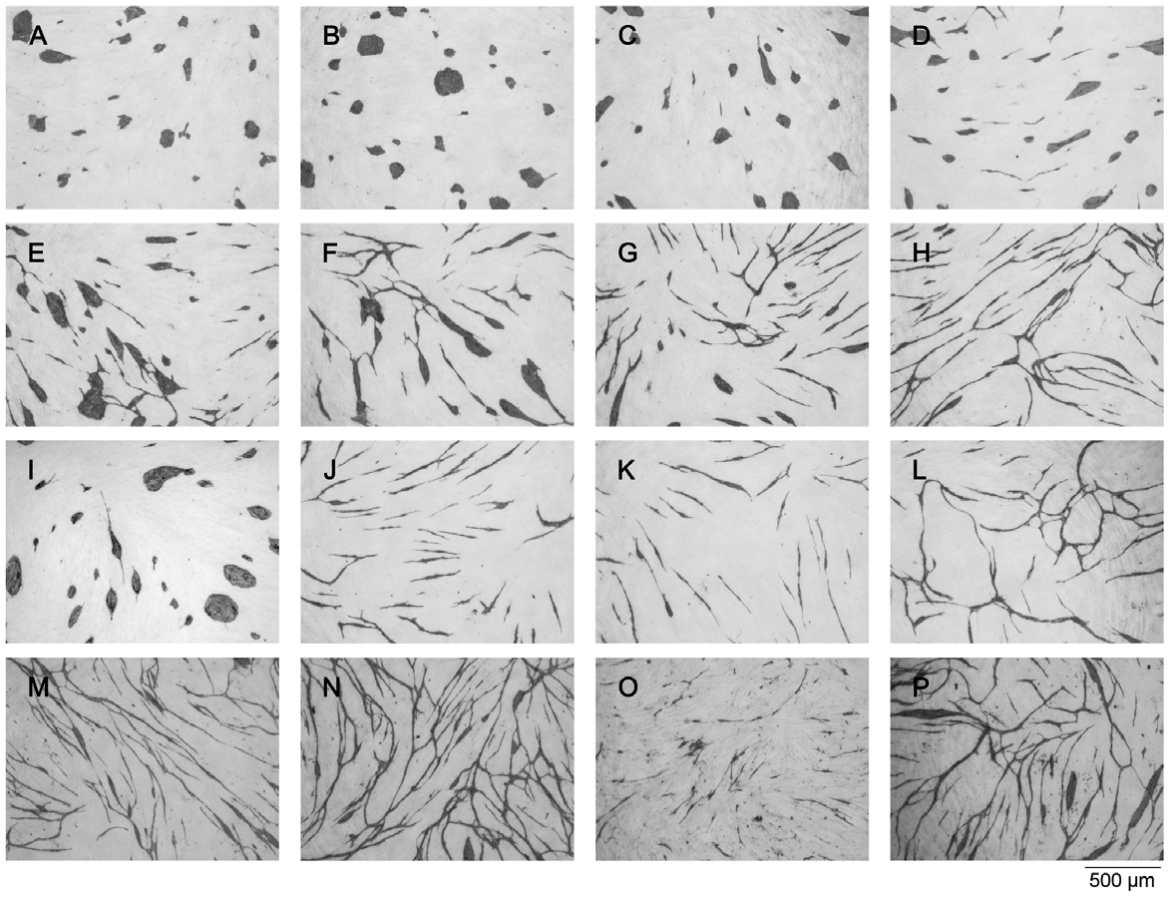
The effect of anti-VEGF and various concentrations of N-methyllevamisole (7a) and suramin (5) recorded in the in vitro angiogenesis assay performed with HUVECs growing on a fibroblast monolayer. The images show HUVECs visualized by immunostaining for CD31 after treatment with: *A)* 1 mM *N*-methyllevamisole triflate (7a); *B)* 0.5 mM *N*-methyllevamisole triflate (**7a**); *C)* 0.25 mM *N*-methyllevamisole triflate (**7a**); *D)* 0.13 mM *N*-methyllevamisole triflate (**7a**); *E)* 0.06 mM *N*-methyllevamisole triflate (**7a**); *F)* 0.03 mM *N*-methyllevamisole triflate (**7a**); *G)* 0.02 mM *N*-methyllevamisole triflate (**7a**); *H)* 0.1 % DMSO (the control was diluted 1∶1000 corresponding the concentration of DMSO present in A); *I)* 5 μg/mL goat anti-recombinant human VEGF; *J)* 12 μM suramin (**5**); *K)* 1.5 μM suramin (**5**); *L)* medium (control); *M)* 10 mM BIS-TRIS; *N)* 10 mM BICINE; *O)* 10 mM *N*-methylimidazole; *P)* 1 mM *N*-methylimidazole.

The antiangiogenic effects of the compounds were determined in a previously developed assay [44], designed to evaluate the growth and differentiation of human umbilical vein endothelial cells (HUVECs) in co-culture with normal human dermal fibroblasts (NHDF). Thus, the effect on differentiation is revealed by HUVEC morphology and ability to form networks while the effect on growth is revealed by HUVEC cell number (Figure 5 and Table 1). The most efficient inhibitors were *p*-bromolevamisole (**3**) (Figure 5C), the aniline-containing dihydro-analogue (**4**) (Figure 5D), and the two different *N*-methyllevamisole salts (**7a, b**) (Figure 5E and F), which were all more potent than (*S*)-levamisole hydrochloride. The racemic tetramisole (**2**) (Figure 5B) had the same effect as the enantiomerically pure parent compound (**1**) (Figure 5A). The cluster morphology known from (*S*)-levamisole, was also observed for tetramisole (**2**) (Figure 5B and Table 1), *p*-bromolevamisole (**3**) (Figure 5C and Table 1), and *N*-methyllevamisoles (**7a, b**), with the latter mentioned showing a slightly more pronounced effect (Figure 5E and F, Figure 6 A–D, Table 1). Whereas the methyl substitution at N-7 seemed to increase the cluster inducing effect relative to compounds **1–3**, the introduction of bulkier groups (**8–12**) still resulted in potent disruption of the network formation, but furnished mixed morphologies containing both cords and clusters (Figure 5G–M and Table 1). Compound **4** (Figure 5D and Table 1), on the other hand, inhibited the capillary network formation to give small cords exclusively, which is reminiscent of the effect of suramin (**5**) (Figure 5N and Table 1), another well-known angiogenesis inhibitor [44], [51].

**Table 1:** Results of in vitro angiogenesis inhibition of levamisole and its derivatives in comparison with suramin (5), vehicle (DMSO), and medium alone, as observed by HUVEC number and morphology.

Compound	Concentration	Area %^a^	Morphology^b^	Grading^c^
**1** (levamisole)	1 mM	7.5	Clusters	++
**2** (tetramisole)	1 mM	8.0	Clusters	++
**3**	1 mM	3.4	Clusters	++++
**4**	1 mM	2.0	Cords	+++
**7a**	1 mM	2.8	Clusters	++++
**7b**	1 mM	3.3	Clusters	++++
**8a**	0.7 mM	5.2	Mixed	+
**8b**	0.2 mM	5.4	Mixed	+
**9**	0.6 mM	2.6	Mixed	+
**10**	0.3 mM	5.0	Mixed	+
**11a**	0.7 mM	2.8	Mixed	+
**11b**	0.4 mM	4.0	Mixed	+
**12**	0.1 mM	3.7	Mixed	+
**5** (suramin) Anti-VEGF	1 mM 5 μg/mL	2.2 8.5	Cords Clusters	+++++
Medium		10.2	Network	
DMSO	Diluted 1:1000	8.4	Network	

a Percent of the area covered by HUVEC as observed by CD31 staining.

b *Clusters* refer to morphologies where several cells that form round or elongated clusters; *Cords* refer to morphologies where single to a few cells form cord-like structures without forming a network.

c (++++)  =  clusters with area <4%; (+++)  =  cords with area <4%; (++)  =  clusters with area >4%; (+)  =  intermediate morphologies and varying area percentages.

Selected compounds were tested with two different counter ions, *i.e., N*-methyllevamisole [triflate (**7a**) and trifluoroacetate (**7b**)], *N*-ethyllevamisole [iodide (**8a**) and trifluoroacetate **(8b**)], and *N*-benzyllevamisole [bromide (**11a**) and trifluoroacetate (**11b**)]. For each of these ion pairs, identical inhibitory effects were observed, strongly indicating that the levamisole analogues rather than the counter ions were responsible for the activities (Figure 5E–H, K, L, and Table 1). *N*-Methyllevamisole triflate (**7a**) showed an IC_50_ for tissue non-specific alkaline phosphatase of 0.1 mM. Since the trifluoroacetate (**7b**) showed no inhibition at 2 mM or higher, however, we speculate that the triflate counter ion may account for the observed discrepancy in this assay.

As mentioned *vide supra* levamisole is an inhibitor of alkaline phosphatase; however, when testing for inhibition of human placenta alkaline phosphatase and tissue non-specific alkaline phosphatase all the derivatives had IC_50_ values of about 1 mM or higher and no correlation to angiogenesis inhibition was therefore observed (Table 2). Since the inhibitory effects in the angiogenesis assay were observed at rather high concentrations of the inhibitors, we also tested various unrelated tertiary amine-containing chemotypes in order to rule out non-specific effects of the positively charged moiety. Figure 6 shows the effect of various concentrations of *N*-methyllevamisole triflate (**7a**) compared to high concentrations (10 mM) of BIS-TRIS, BICINE, and *N*-methylimidazole, respectively. Neither BIS-TRIS nor BICINE showed any effects even at 10 mM (Figure 6M–N), while titration experiments showed that *N*-methyllevamisole triflate (**7a**) exerted its cluster-inducing effect already at 30 μM (Figure 6F), which is closer to a physiologically relevant concentration than the 1 mM concentration used in the initial screen. *N*-methylimidazole had an effect at 10 mM (Figure 6O), but no effect at 1 mM (Figure 6P). Other amine-containing compounds (HEPES, TRICINE and TRIZMA) neither showed an effect in the assay (results not shown).

**Table 2:** Inhibition of human placenta alkaline phosphatase (HPAP), bovine kidney tissue non-specific alkaline phosphatase (TNAP) and sirtuin 1 (SIRT1) by selected compounds.

Compound	1	2	3	5	7a	7b	8a	9	11a	11b	12
**HPAP IC_50_ (mM)**	1.1	1.2	0.7	ND	>2	>2	>2	>2	>2	>2	>2
**TNAP IC_50_ (mM)**	0.6	1.0	>2	>2	0.1	>2	>2	>2	>2	>2	>2
**SIRT1 inhib. (1 μM)** a	ND	ND	ND	>90%	Not active*^b^*	ND	ND	ND	ND	ND	ND

a Sirtuin 1 inhibition was tested for *N*-methyllevamisole (**7a)** and suramin (**5**) as a positive control. *^b^* No inhibition was observed at ligand concentrations up to 10 mM. All assays were performed at least twice in duplicate. ND  =  not determined.

As previously described [38], we show that antibodies targeting vascular endothelial growth factor (VEGF) in a concentration of 5 μg/mL inhibit endothelial cell tube formation in the co-culture assay due to its VEGF neutralizing effect, and as a result induce the same cluster morphology as *N*-methyllevamisole triflate (**7a**) (Figure 6 A–D and I). Notably, this effect is identical to what is observed in the assay when VEGF is omitted from the growth medium [37], [38], which may indicate that *N*-methyllevamisole triflate (**7a**) effectively interferes with VEGF signalling, albeit at relatively high concentrations. Though, based on this preliminary test, we cannot rule out possible interaction of these chemotypes with other phosphatases and/or kinases. In a recent publication, several phosphatase inhibitors were shown to exhibit angiogenesis inhibitory activity [52].

**Figure 7:**
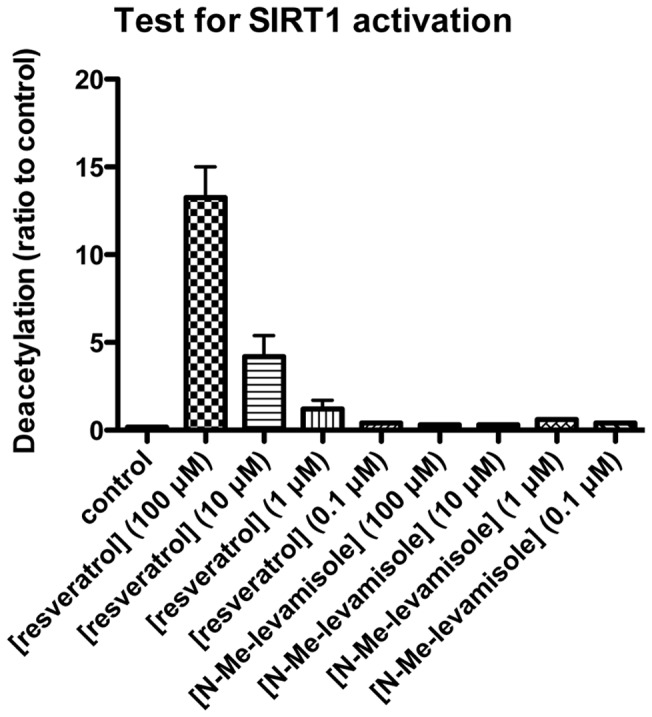
Test for activation of sirtuin 1 (SIRT1). The ability of compound **7a** to activate SIRT1 mediated deacetylation was tested in a commercially available fluorogenic assay. Resveratrol was used a positive control, although its relevance as a direct activator of sirtuin activity has been called into question recently [59]–[62]. The data represent two individual assays performed in duplicate.

Finally, we checked compound **7a** for its ability to inhibit sirtuin 1 (SIRT1) deacetylase activity in a standard fluorogenic assay *in vitro*, since suramin is a known SIRT1 inhibitor [53], and SIRT1 has been shown to be a regulator of angiogenesis [54]. As expected suramin potently inhibited SIRT1, but compound **7a** exhibited no inhibitory effect (Table 2). Rather curiously, however, elevated values of deacetylation compared to the control wells were observed at 10 μM and 100 μM (data not shown). Thus, to make sure whether or not this was an artifact in the assay, we also tested **7a** for its ability to activate SIRT1 compared to resveratrol, by following standard protocols given by the supplier. These experiments ruled out interaction with this regulatory enzyme (Figure 7).

Intracellular signaling is mediated through several pathways, including phosphorylation and dephosphorylation cascades, which are of major importance [55], [56]. This also applies to VEGF signaling, which comprises several phosphorylation events, including receptor (auto) phosphorylation [19]. Consequently, protein kinases are potential targets for interfering with all aspects of cell growth including angiogenesis [57]. Since kinases and phosphatases share many substrate features, their inhibitors may also exhibit considerable overlap in specificity within the kinase and the phosphatase families. Therefore, levamisole and its novel derivatives described herein may potentially influence both kinases and phosphatases in the VEGF signaling pathway, although data to support this hypothesis is still required to draw conclusions regarding the mechanism of action. Interestingly, however, levamisole was recently included in a large investigation describing the effects of known drugs (107 compounds) on a variety of biochemical pathways in cells by protein-fragment complementation assays (PCAs; 49 included) [58]. Among others, levamisole showed effects on Bcl-associated death promoter (BAD), Bcl-xL, p21, mitogen-activated protein kinase 2 (MAPK2), and LIM domain kinase 2 (Limk2), which may serve as inspiration in the future search for the putative target of levamisole and its novel analogues. Furthermore, it was notable, that levamisole's closest neighbors in a clustering analysis based on those results were the NSAID celecoxib (also used to treat rheumatoid arthritis) and rosuvastatin (an inhibitor of HMG-CoA-reductase, which is natively regulated by phosphorylation) [58].

In summary, a series of derivatives of levamisole were evaluated for their ability to inhibit angiogenesis *in vitro*, which revealed several compounds that disrupted network formation and gave rise to different morphological phenotypes. One group, including *p*-bromolevamisole and tetramisole induced cluster formation similar to that observed for levamisole, while *N-*methyllevamisole was more efficient than the parent inhibitor and gave rise to a slightly different morphology. The effect of an aniline-containing analogue (**4**) resembled that of suramin by furnishing network inhibition with small cord morphology, albeit at considerably higher concentrations. The third group, which comprised the majority of the N-alkylated analogues, were all inhibitors of network formation giving rise to mixed morphologies. It should be emphasized that both (*S*)-levamisole and the two derivatives (**3**) and (**7a**) are not considered to be potent inhibitors, although the cluster inducing effect of **7a** was demonstrated down to 30 μM. Since these compounds represent a novel scaffold for angiogenesis inhibitors, and since the morphology observed after treatment with *N*-methyllevamisole (**7a**) differs from that of suramin and levamisole, we find that this novel class of chemotypes warrant further investigation.

Future work will focus on gaining a deeper understanding of the effects of (*S*)-levamisole and (*S*)-*N*-methyllevamisole on intracellular signaling, as well as the mechanisms responsible for differentiation and growth of endothelial cells. Development of more potent derivatives exhibiting a similar morphological effect is of great interest, but because levamisole is approved for clinical use, *in vivo* efficacy and toxicology profiling of the novel N-methylated inhibitor will also be an objective.

## Materials and Methods

### General

All reagents and solvents from commercial suppliers were used without further purification. Alkaline phosphatase-conjugated goat anti-mouse IgG, BICINE, BIS-TRIS, bovine kidney alkaline phosphatase, 5-bromo-4-chloro-3-indolyl phosphate/nitro blue tetrazolium tablets (BCIP/NBT), *p*-nitrophenyl phosphate substrate tablets (*p*-NPP), and suramin were from Sigma (St. Louis, USA). Levamisole, *N*-methylimidazole and tetramisole were from Aldrich (Milwaukee, WI, USA), (–)-*p*-bromolevamisole oxalate was from Sigma-Aldrich (Schnelldorf, Germany), and 4-(2,3-dihydroimidazo-[2,1-b]thiazol-6-yl)aniline was from Maybridge (Trevillett, UK). Human serum albumin (HSA) and TTN buffer (0.05 M Tris, pH 7.5, 0.3 M NaCl, 1% v/v Tween 20) were from Statens Serum Institut (Copenhagen, Denmark). Ethanol (96%) was from Danisco (Aalborg, Denmark). Acetic acid, acetone, DMSO, Na_2_HPO_4_, NaH_2_PO_4_, Tween 20, NaCl, HPLC-grade acetonitrile, and formic acid were from Merck (Darmstadt, Germany). Mouse anti-human CD31 was from Monosan (Uden, Netherlands). Goat anti-recombinant human vascular endtothelial growth factor (Anti-VEGF), recombinant basic fibroblast growth factor (bFGF) and recombinant human vascular endothelial growth factor (VEGF) were from R&D Systems (Minneapolis, MN, USA). Human umbilical vein endothelial cells (HUVEC), normal human dermal fibroblast (NHDF), the HUVEC media-kit EGM-2 Bulletkit, and the fibroblast media-kit FGM-2 Bulletkit were from Clonetics, BioWhittaker (Walkersville, MD, USA). Polystyrene 96-microwell plates were from Thermo Fischer Scientific (Roskilde, Denmark). Trifluoroacetic acid (TFA) was from Rathburn (Walkerburn, Scotland, UK). Porous column material was from Applied Biosystems (Foster City, California, USA). PBS was made from 8 mM Na_2_HPO_4_, 2 mM NaH_2_PO_4_, 0.15 M NaCl, pH 7.3. ^1^H and ^13^C NMR spectra were recorded on a Bruker AMX 400 instrument and are reported in *δ* units (ppm). The solvent peak (CDCl_3_ or CD_3_OD) was used as internal reference. Values of coupling constants *J* are given in Hz and the signal multiplicities are shown in parentheses (singlet (s), doublet (d), triplet (t), quartet (q), heptet (h), multiplet (m)). For an example (compound **12**) of a full assignment of the signals, see Table S1. Optical rotations were measured on a 241 polarimeter (Perkin Elmer, Waltham, Massachusets, USA). Vacuum liquid chromatography (VLC) was performed with silicagel 60 H (particle size <45 μm). Preparative reversed phase HPLC separations were carried out on a Phenomenex Luna 250×21.2 mm, C18 column (5 μm) using an Agilent system consisting of two preparative scale pumps, an autosampler, and a multiple-wavelength UV detector. A gradient elution systems consisting of eluent A (H_2_O–MeCN–TFA 95∶5∶0.1) and eluent B (H_2_O–MeCN–TFA 5∶95∶0.1), rising linearly from 0% to 30% of B during 25 min was used. The eluent flow rate was maintained at 20 mL/min and injection volumes were 900 μL. HRMS was performed by Ultra High Performance Liquid chromatography-high resolution mass spectrometry (UHPLC-HRMS) on a maXis G3 quadrupole time-of-flight mass spectrometer (Bruker Daltonics, Bremen, Germany) equipped with an electrospray (ESI) source. The MS was connected to an Ultimate 3000 UHPLC system (Dionex, Sunnyvale, CA). Separation of 1 µL samples were performed at 40°C on a 100 mm ×2.1 mm, 2.6 µm Kinetex C_18_ column (Phenomenex, Torrance, CA) using a linear water-acetonitrile gradient (both buffered with 20 mM formic acid) at a flow of 0.4 mL min^−1^ starting from 10% acetonitrile and increased to 100% over 10 minutes. MS was performed in ESI^+^ with a data acquisition range of 10 scans per sec at *m/z* 100–1000. The MS was calibrated using sodium formate automatically infused prior to each analytical run, providing a mass accuracy of less than 0.5 ppm in MS mode.

### (*S*)-Levamisole free base (6) [50]

Levamisole hydrochloride (2.00 g, 8.31 mmol) was suspended in Et_2_O (50 mL) and concentrated aqueous NaOH (10 mL, 35%) and H_2_O (15 mL) were added. The mixture was extracted with Et_2_O (4×20 mL) and the combined Et_2_O extracts were dried (Na_2_SO_4_), filtered, concentrated *in vacuo*, and dried under high vacuum with an oil pump overnight. The levamisole (1.68 g, 99%) was obtained as a colorless syrup, which was used without further purification. ^1^H NMR (400 MHz, 298.2 K, CD_3_OD): δ = 3.00 (dd, 1H, *J*_5α, 6_ = 9.2, *J*_5α, 5β_  = 8.6, H-5_α_), 3.18 (m, 1H, AB part of a larger spin system, 2-H_A/B_), 3.41 (ddd, 1H, *J*_3α, 3β_  = 11.0, *J*_3α, 2A/B_  = 6.6, *J*_3α, 2A/B_  = 4.5, H-3_α_), 3.61 (ddd, 1H, *J*_3β, 3α_  = 11.0, *J*_3β, 2A/B_  =  6.6, *J*_3β, 2A/B_  = 4.5, H-3_β_), 3.69 (m, 1H, A/B part of a larger spin system, 2-H_A/B_), 3.72 (dd, 1H, *J*_5β, 6_  = 8.7, *J*_5β, 5α_  = 8.6, H-5_β_), 5.38 (t, 1H, *J*_6, 5β/5α_  = 9.0, H-6), 7.32 (m, 5H, H-Ar). ^13^C NMR (100 MHz, 298.2 K, CD_3_OD): δ = 35.36 (C-3), 50.23 (C-2), 59.37 (C-5), 77.52 (C-6), 127.83, 129.68 (4C, C-2′, C-3′, C-5′, C-6′), 128.65 (C-4′), 144.15 (C-1′), 177.49 (C-8).

### (*S*)-*N*-Methyllevamisole (7a and 7b)

Levamisole (0.233 g, 1.14 mmol) was dissolved in Et_2_O (10 mL) and stirred under nitrogen atmosphere. Methyltriflate (0.164 mL, 0.24 mmol) was added and the mixture stirred for 1 h. The reaction mixture was evaporated to dryness, and pure *N*-methyllevamisole triflate (**7a**) was obtained as syrup after lyophilization (>95% purity by ^1^H NMR). From this crude product, 161 mg was purified by reversed phase HPLC to give *N*-methyllevamisole trifluoroacetate (**7b**) (81 mg, 57%). Optical rotation (**7a**): [α]_D_ –83° (*c* 0.01, 298.2 K, CH_2_Cl_2_), lit. [α]_D_ –102° (ref [50]). ^1^H NMR (400 MHz, 298.2 K, CD_3_OD) the data were identical for **7a** and **7b**, and agreed with the previously published results [50]: δ = 2.90 (s, 3H, H-1′′), 3.75 (dd, 1H, *J*_5α, 5β_  = 10.4, *J*_5α, 6_  = 9.1, H-5_α_), 3.90 (m, 2H, A/B part of a larger spin system, H-2), 4.09 (m, 2H, A/B part of a larger spin system, H-3), 4.27 (t, 1H, *J*_5β, 5α/6_  = 10.4, H-5_β_), 5.56 (dd, 1H, *J*_6, 5β_  = 10.4, *J*_6, 5α_  = 9.1, H-6), 7.49 (m, 5H, H-Ar). ^13^C NMR (100 MHz, 298.2 K, MeOD): δ = 34.0 (C-1′′), 38.1 (C-3), 50.2 (C-2), 56.1 (C-5), 74.5 (C-6), 129.0, 130.8 (4C, C-2′, C-3′, C-5′, C-6′), 131.2 (C-4′), 136.9 (C-1′′), 179.3 (C-8). MS (*m/z*): [M]^+^ calcd, 219.1; found, 219.3.

### General procedure I; alkylation of the free base of *(S)*-levamisole (6) with alkyl halides

Levamisole (**6**) (about 1.2 mmol) was dissolved in THF (5 mL) and stirred under a nitrogen atmosphere. The proper iodo- or bromoalkyl reagent (1.2 equiv) was added, and the solution was stirred under reflux for 24 h. Then the mixture was cooled to room temperature and concentrated *in vacuo*, redissolved in H_2_O (25 mL), and washed with CH_2_Cl_2_ (4×25 mL). The aqueous phase was concentrated under reduced pressure, flash frozen, and lyophilized to give the desired *N*-alkyl-levamisole halide, which was tested directly and/or purified by reversed phase HPLC to give its corresponding trifluoroacetate salt.

### (*S*)-*N*-Ethyllevamisole (8a and 8b)

Levamisole (0.252 g, 1.24 mmol) and iodoethane (0.123 mL, 1.54 mmol) were subjected to general procedure I, which furnished the iodide (**8a**) after lyophilization. A sample of the colorless syrup was taken up in MeOH (0.9 mL) and purified by reversed phase HPLC to give the *N*-ethyllevamisole trifluoroacetate (**8b**) (129 mg, 30%). Optical rotation (**8b**): [α]_D_ –73° (*c* 0.01, 298.2 K, CH_2_Cl_2_). ^1^H NMR (400 MHz, 298.2 K, CD_3_OD) the data were identical for **8a** and **8b**: δ = 1.12 (t, 3H, *J*_2′′, 1′′_  = 7.3, H-2′′), 3.20 (dq, 1H, *J*_1′′α, 1′′β_  = 14.4, *J*_1′′α, 2′′_  = 7.3, H-1′′_α_), 3.35 (dq, 1H, *J*_1′′β, 1′′α_  = 14.4, *J*_1′′α, 2′′_  = 7.3, H-1′′_α_), 3.77 (dd, 1H, *J*_5α, 5β_  = 10.4, *J*_5α, 6_ = 8.8, H-5), 3.89 (m, 2H, A/B part of a larger spin system, H-2), 4.09 (m, 2H, A/B part of a larger spin system, H-3), 4.27 (t, 1H, *J*_5β, 5α/6_  = 10.4, H-5_β_), 5.69 (dd, 1H, *J*_6, 5β_  = 10.4, *J*_6, 5α_  = 8.8, H-6), 7.50 (m, 5H, H-Ar). ^13^C NMR (100 MHz, 298.2 K, CD_3_OD): δ = 12.7 (C-2′′), 38.0 (C-3), 43.5 (C-1′′), 49.9 (C-2), 56.2 (C-5), 72.3 (C-6), 129.2, 130.8 (4C, C-2′, C-3′, C-5′, C-6′), 131.3 (C-4′), 137.1 (C-1′), 178.5 (C-8). MS (*m/z*): [M]^+^ calcd, 233.1; found, 233.3. HRMS (*m/z*): [M]^+^ calcd for C_13_H_17_N_2_S, 233.1107; found, 233.1107 (ΔM, 0.2 ppm).

### (*S*)-*N*-Isopropyllevamisole trifluoroacetate (9)

Levamisole (0.224 g, 1.37 mmol) and 2-iodopropane (0.137 mL, 1.37 mmol) were subjected to general procedure I, with stirring under reflux for 3 days. Purification by reversed phase HPLC provided the title compound as a colorless syrup (53 mg, 13%). Optical rotation: [α]_D_ –70° (*c* 0.01, 298.2 K, CH_2_Cl_2_). ^1^H NMR (400 MHz, 298.2 K, CD_3_OD): δ = 1.11 (d, 3H, *J*_2′′A/B, 1′′_  = 6.8, H-2′′_AB_), 1.28 (d, 3H, *J*_2′′AB, 1′′_  = 6.8, H-2′′_AB_), 3.63 (h, 1H, *J*_1′′, 2′′A/B_  = 6.8, H-1′′), 3.75 (dd, 1H, *J*_5α, 5β_  = 10.4, *J*_5α, 6_ = 8.3, H-5_α_), 3.86 (m, 2H, A/B part of a larger spin system, H-2), 4.07 (m, 2H, A/B part of a larger spin system, H-3), 4.24 (t, 1H, *J*_5β, 5α/6_ = 10.4, H-5_β_), 5.70 (dd, 1H, *J*_6, 5β_  = 10.4, *J*_6, 5α_  = 8.3, H-6), 7.50 (m, 5H, H-Ar). ^13^C NMR (100 MHz, 298.2 K, CD_3_OD): δ = 20.3 (C-2′′_A/B_), 20.9 (C-2′′_A/B_), 38.0 (C-3), 49.2 (C-2), 52.8 (C-1′′), 56.3 (C-5), 72.3 (C-6), 129.1 and 130.6 (4C, C-2′, C-3′, C-5′, C-6′), 131.1 (C-4′), 138.5 (C-1′), 177.2 (C-8). MS (*m/z*): [M]^+^ calcd, 247.1; found, 247.3. HRMS (*m/z*): [M]^+^ calcd for C_14_H_19_N_2_S, 247.1263; found, 247.1263 (ΔM, 0.1 ppm).

### (*S*)-*N*-(*n*-Butyl)-levamisole trifluoroacetate (10)

Levamisole (0.252 g, 1.24 mmol) and 1-iodobutane (0.176 mL, 1.54 mmol) were reacted according to general procedure I to give the title compound as a colorless syrup (40 mg, 9%) after reversed phase HPLC purification. Optical rotation: [α]_D_ –59° (*c* 0.01, 298.2 K, CH_2_Cl_2_). ^1^H NMR (400 MHz, 298.2 K, CD_3_OD): δ = 0.86 (t, 3H, *J*_4′′, 3′′_  = 7.2, H-4′′), 1.28 (m, 2H, H-3′′), 1.47 (m, 2H, H-2′′), 3.14 (dt, 1H, *J*_1α'', 1′′β_  = 14.5, *J*_1α'', 2′′_  = 6.7, H-1′′_α_), 3.27 (dt, 1H, *J*_1′′β, 1α''_  = 14.5, *J*_1′′β, 2′′_  = 6.7, H-1′′_β_), 3.78 (dd, 1H, *J*_5α, 5β_  = 10.5, *J*_5α, 6_ = 8.9, H-5_α_), 3.90 (m, 2H, A/B part of a larger spin system, H-2), 4.09 (m, 2H, A/B part of a larger spin system, H-3), 4.28 (t, 1H, *J*_5β, 5α/6_ = 10.5, H-5_β_), 5.66 (dd, 1H, *J*_6, 5β_  = 10.5, *J*_6, 5α_  = 8.8, H-6), 7.50 (m, 5H, H-Ar). ^13^C NMR (100 MHz, 298.2 K, CD_3_OD): δ = 13.9 (C-4′′), 20.8 (C-3′′), 30.2 (C-2′′), 38.0 (C-3), 48.4 and 50.0 (2C, C-2, C-1′′), 56.1 (C-5), 72.7 (C-6), 129.3, 130.8 (4C, C-2′, C-3′, C-5′, C-6′), 131.3 (C-4′), 137.0 (C-1′), 178.9 (C-8). MS (*m/z*): [M]^+^ calcd, 261.1; found, 261.3. HRMS (*m/z*): [M]^+^ calcd for C_15_H_21_N_2_S, 261.1419; found, 261.1420 (ΔM, 0.1 ppm).

### (*S*)-*N*-Benzyllevamisole (11)

Levamisole (0.290 g, 1.42 mmol) was dissolved in THF (5 mL) and benzylbromide (0.210 mL, 1.78 mmol) were subjected to general procedure I, which furnished the bromide (**11a**) after lyophilization. A sample (48 mg, 0.14 mmol) of the colorless syrup was taken up in MeOH (1 mL) and purified by reversed phase HPLC to give the *N*-benzyllevamisole trifluoroacetate (**11b**; 10 mg). Optical rotation (**11b**): [α]_D_ –46° (*c* 0.01, 298.2 K, CH_2_Cl_2_). ^1^H NMR (400 MHz, 298.2 K, CD_3_OD): δ = 3.80 (dd, 1H, *J*_5α, 5β_  = 10.5, *J*_5α, 6_ = 9.3, H-5_α_), 3.90 (m, 2H, A/B part of a larger spin system, H-2), 4.04 (m, 2H, A/B part of a larger spin system, H-3), 4.23 (d, 1H, *J*_1′′β, 1′′α_  = 15.1, H-1′′_β_), 4.27 (t, 1H, *J*_5β, 5α/6_ = 10.5, H-5 _β_), 4.46 (d, 1H, *J*_1′′α, 1′′β_  = 15.1, H-1′′_α_), 5.52 (dd, 1H, *J*_6, 5β_  = 10.5, *J*_6, 5α_  = 9.3, H-6), 7.16 (m, 2H, H-3′′, H-7′′), 7.35 (m, 3H, H-4′′, H-5′′, H-6′′), 7.48 (m, 5H, H-Ar). ^13^C NMR (100 MHz, 298.2 K, CD_3_OD): 38.0 (C-3), 50.0 (C-2), 52.0 (C-1′′), 55.9 (C-5), 72.9 (C-6), 129.4, 130.2, and 130.7 (8C, C-2′, C-3′, C-5′, C-6′, C-3′′, C-4′′, C-6′′, C-7′′), 130.3 (2C, C-4′, C-5′′), 133.5 (C-2′′), 136.5 (C-1′), 178.7 (C-8). MS (*m/z*): [M]^+^ calcd, 295.1; found, 295.4.

### (*S*)-*N*-Cyclohexylmethylenelevamisole (12)

Levamisole (**6**) (0.149 g, 0.73 mmol) and cyclohexyl-bromomethane (0.126 mL, 0.91 mmol) were reacted according to general procedure I, purified by reversed phase HPLC, and lyophilized to give **12** as the trifluoroacetate (128 mg, 22%). Optical rotation: [α]_D_ –46° (*c* 0.01, 298.2 K, CH_2_Cl_2_). ^1^H NMR (400 MHz, 298.2 K, CD_3_OD): δ = 0.86 (m, 2H, H-5′′), 1.16 (m, 2H, H-4′′), 1.15 (m, 1H, H-3′′B), 1.46 (m, 1H, H-2′′), 1.54 (m, 1H, H-7′′A), 1.55 (m, 1H, H-3′′A), 1.67 (m, 1H, H-7′′B), 1.69 (m, 2H, H-6′′), 2.96 (dd, 1H, *J*_1′′α, 1′′β_  = 14.4, *J*_1′′α, 2′′_  = 6.29, H-1′′_α_), 3.07 (dd, 1H, *J*_1′′β, 1′′α_  = 14.4, *J*_1′′β, 2′′_  = 8.44, H-1′′_β_), 3.80 (dd, 1H, *J*_5α, 5β_  = 10.4, *J*_5α, 6_  = 8.8, H-5_α_), 3.87 (m, 1H, A/B part of a larger spin system, H-2_A/B_), 3.93 (m, 1H, A/B part of a larger spin system, H-2_A/B_), 4.08 (m, 1H, A/B part of a larger spin system, H-3_A/B_), 4.10 (m, 1H, AB part of a larger spin system, H-3_A/B_), 4.29 (t, 1H, *J*_5β, 5α/6_ = 10.4, H-5_β_), 5.66 (dd, 1H, *J*_6, 5β_  = 10.4, *J*_6, 5α_  = 8.8, H-6), 7.48 (m, 1 H, H-4′), 7.49 (m, 2 H, H-3′ ), 7.51 (m, 2 H, H-2′). ^13^C NMR (100 MHz, 298.2 K, CD_3_OD): δ = 26.5 (C-6′′), 26.6 (C-3′′), 27.1 (C-4′′), 31.5 (C-7′′), 31.6 (C-5′′), 37.1 (C-2′′), 37.8 (C-3), 49.9 (C-2), 54.9 (C-1′′), 55.9 (C-5), 73.0 (C-6), 129.2 (2C, C-2′, C-6′), 130.7 (2C, C-3′, C-5′), 131.3 (C-4′), 136.9 (C-1′), 179.1 (C-8). MS (*m/z*): [M]^+^ calcd, 301.1; found, 301.4. HRMS (*m/z*): [M]^+^ calcd for C_18_H_25_N_2_S, 301.1733; found, 301.1733 (ΔM, 0.1 ppm).

### Endothelial cell culture

Co-culture cell assays were performed according to a method described previously [38], [44]. Briefly, 10^3^ NHDFs were cultured in each well of a 96-well microtiter plate in NHDF standard medium (100 μL, FGM-2 Bullet kit) at 37°C in an atmosphere containing 5% CO_2_. After 3 days the medium was removed and 10^3^ HUVECs were seeded onto the NHDF in TFSM2 medium (135 μL). The samples of the individual compounds were prepared by weight and dissolved in a concentration of 1 M in DMSO, PBS, or Milli-Q water followed by pH adjustment, and sterile filtration through 0.22 μm filters. Medium, buffer controls or test samples of 15 µL were added to each well containing the HUVEC–NHDF co-culture and were incubated for 3 days. Experiments were repeated 2–4 times with identical results.

### CD31 immunostaining

HUVEC and fibroblasts were stained with a CD31 antibody according to the method previously described [38], [44]. Briefly, the cells were washed with PBS, fixed with ethanol, blocked with TTN buffer, incubated with mouse anti-human CD31 diluted 1∶250 in TTN buffer, washed in TTN buffer and incubated with alkaline phosphatase-conjugated goat anti-mouse IgG diluted 1∶1000 in TTN buffer. Bound antibody was subsequently visualised with BCIP/NBT and inspected in an Olympus IX70 microscope and photographed with an Olympus DP12 digital camera (Magnification: 40x). Image analysis was carried out using the program AnalySIS (Soft Imaging Systems, Münster, Germany).

### Alkaline phosphatase assay

The levamisole derivatives were diluted to 2.5, 5 and 10 mM in DMSO. Samples (10 µL) and 90 µL solution of alkaline phosphatase (0.01 U/mL) from human placenta or bovine kidneys diluted in substrate buffer were added to a 96-well microtiter plate and incubated for 35 minutes at room temperature. Hereafter, 100 µL *p*-nitrophenyl phosphate (2 mg/mL) were added, and the plate was read at 405 nm with background subtraction at 650 nm on a Versamax Elisa reader (Molecular Devices, Sunnyvale, CA, USA) after 24 and 35 min. Results were obtained as the mean of double determinations. The EC_50_ values were read from the curve at half maximal absorbance.

### SIRT1 inhibition assay

The assay was performed according to protocols provided by the supplier (Biomol, BML-AK555). Briefly, the dose–response experiments were performed in black low binding NUNC 96-well microtiter plates. The dilution series were prepared in Milli-Q water from 100 mM DMSO stock solutions. Fluor-de-Lys SIRT1 substrate (250 μM), NAD^+^ (500 M), and SIRT1 (2 U/well) were incubated in SIRT assay buffer prepared as described in the Biomol product sheets [Tris/Cl (50 mM), NaCl (137 mM), KCl (2.7 mM), MgCl_2_ (1 mM), pH 8.0, 1 mg/mL bovine serum albumin] in the presence or absence of the appropriate dilution of compound **7a** or suramin. After 1 hour at 37°C, nicotinamide (2 mM) and developer (50 μL, 2×) were added, and the assay development was allowed to proceed for 15–30 minutes at room temperature, before the plate was read using a Tecan plate reader with excitation at 360 nm and emission at 460 nm. Two individual assays were performed in duplicate.

### SIRT1 activation assay

The assay was essentially performed as described above for the deacetylase inhibition, but with different concentrations of enzyme and substrate [Fluor-de-Lys SIRT1 substrate (25 μM), NAD^+^ (25 μM), SIRT1 (1 U/well)], and using resveratrol as positive control. Two individual assays were performed in duplicate.

## Supporting Information

Table S1 **Full assignment of the NMR spectral data obtained for compound 12.**
(PDF)
