# Supplementary material for: Synthesis and Antiangiogenic Activity of N-Alkylated Levamisole Derivatives
Source: PLoS One. 2012 Sep 14;7(9):e45405. doi: 10.1371/journal.pone.0045405 (PMC3443238; doi:10.1371/journal.pone.0045405)
Supplement: Table S1 — Full assignment of the NMR spectral data obtained for compound 12. (PDF) [file pone.0045405.s001.pdf]

Supporting Information

Synthesis and Antiangiogenic Activity of N-Alkylated Levamisole Derivatives

Anders N. Hansen[1,#], Christine D. Bendiksen[2,#], Lene Sylvest[2], Tina Friis[2], Dan Staerk[1,3], Flemming S. Jørgensen[1], Christian A. Olsen[1,4,]*, and Gunnar Houen[2,]*

* E-mails: cao@kemi.dtu.dk (CAO) and gh@ssi.dk (GH).

<u>Contents:</u>

Page S2–S9; LC-MS data.

Page S10–S36; ^{1}H NMR, ^{13}C NMR, and DEPT 135 spectra.

(**7a** S10–12, **7b** S13–15, **8a** S16–18, **8b** S19–21, **9** S22–24, **10** S25–27, **11a** S28–30, **11b** S31–33, and **12** S34–36)

Page S37; Table S1.

Compound **7a**

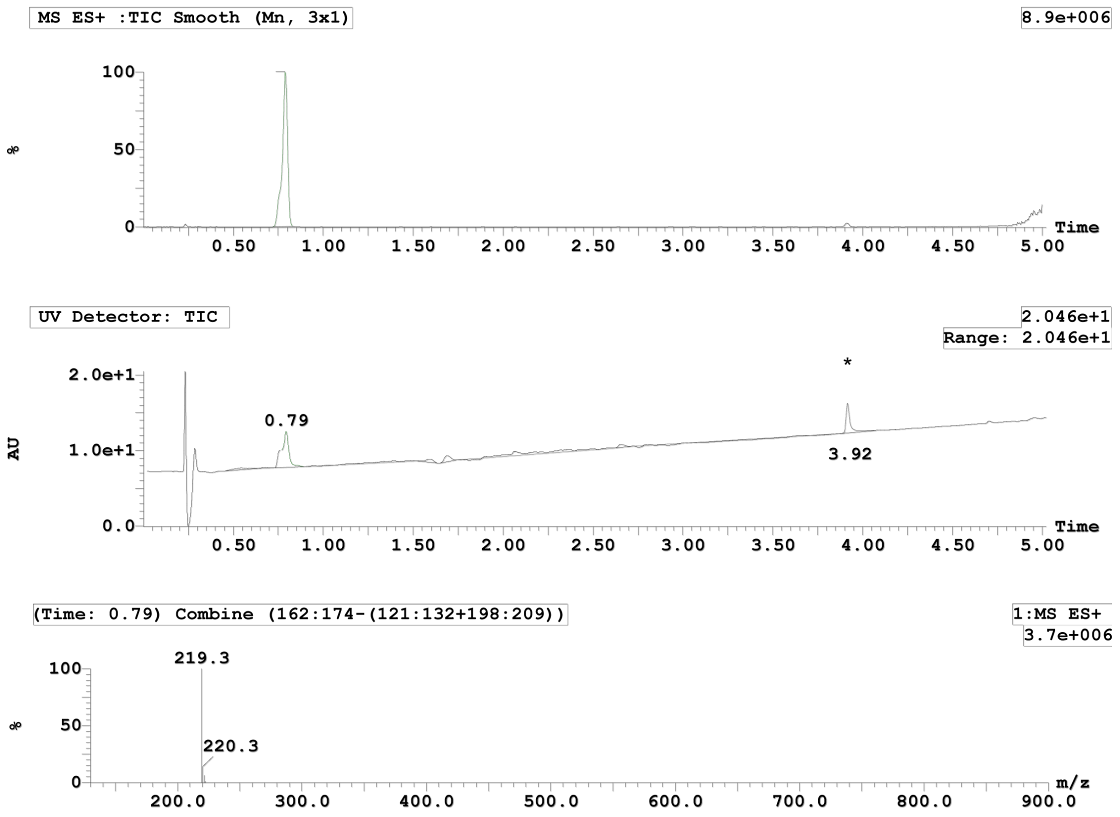

*Ghost peak from the UPLC-MS system.

Compound **7b**

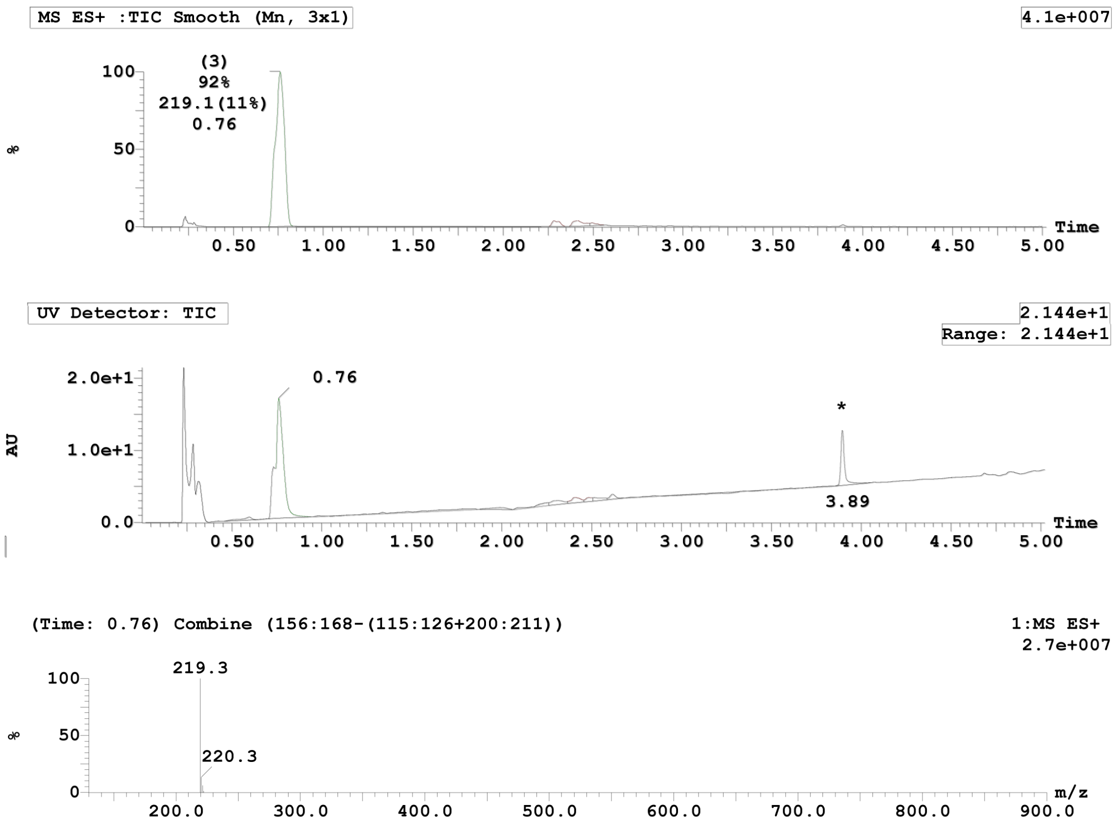

MS ES+ :TIC Smooth (Mn, 3x1)
4.1e+007
(3)
92%
219.1(11%)
0.76
100
50
0
%
Time
0.50 1.00 1.50 2.00 2.50 3.00 3.50 4.00 4.50 5.00
UV Detector: TIC
2.144e+1
Range: 2.144e+1
2.0e+1
1.0e+1
0.0
AU
0.76
*
3.89
Time
0.50 1.00 1.50 2.00 2.50 3.00 3.50 4.00 4.50 5.00
(Time: 0.76) Combine (156:168-(115:126+200:211))
1:MS ES+
2.7e+007
219.3
220.3
100
50
0
%
m/z
200.0 300.0 400.0 500.0 600.0 700.0 800.0 900.0

Compound **8a**

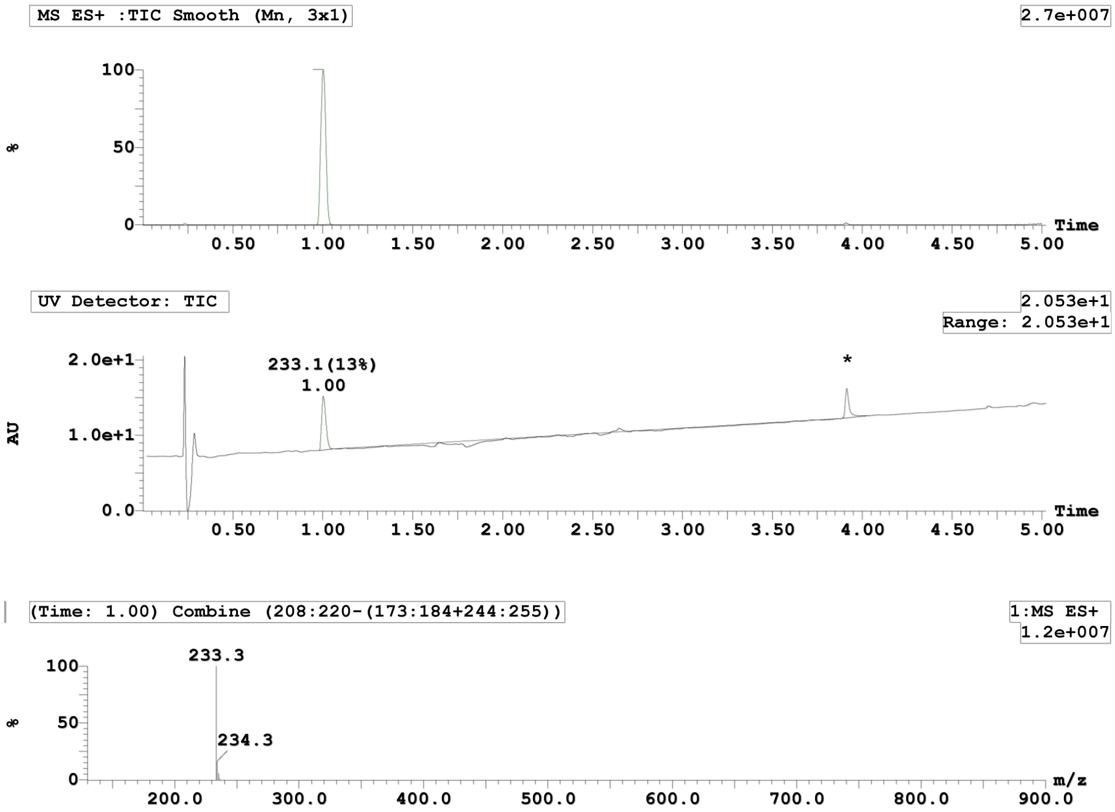

MS ES+ :TIC Smooth (Mn, 3x1)
2.7e+007
100
50
0
%
Time
0.50 1.00 1.50 2.00 2.50 3.00 3.50 4.00 4.50 5.00
UV Detector: TIC
2.053e+1
Range: 2.053e+1
2.0e+1
1.0e+1
0.0
AU
233.1(13%)
1.00
*
Time
0.50 1.00 1.50 2.00 2.50 3.00 3.50 4.00 4.50 5.00
(Time: 1.00) Combine (208:220-(173:184+244:255))
1:MS ES+
1.2e+007
233.3
234.3
100
50
0
%
m/z
200.0 300.0 400.0 500.0 600.0 700.0 800.0 900.0

Compound **9**

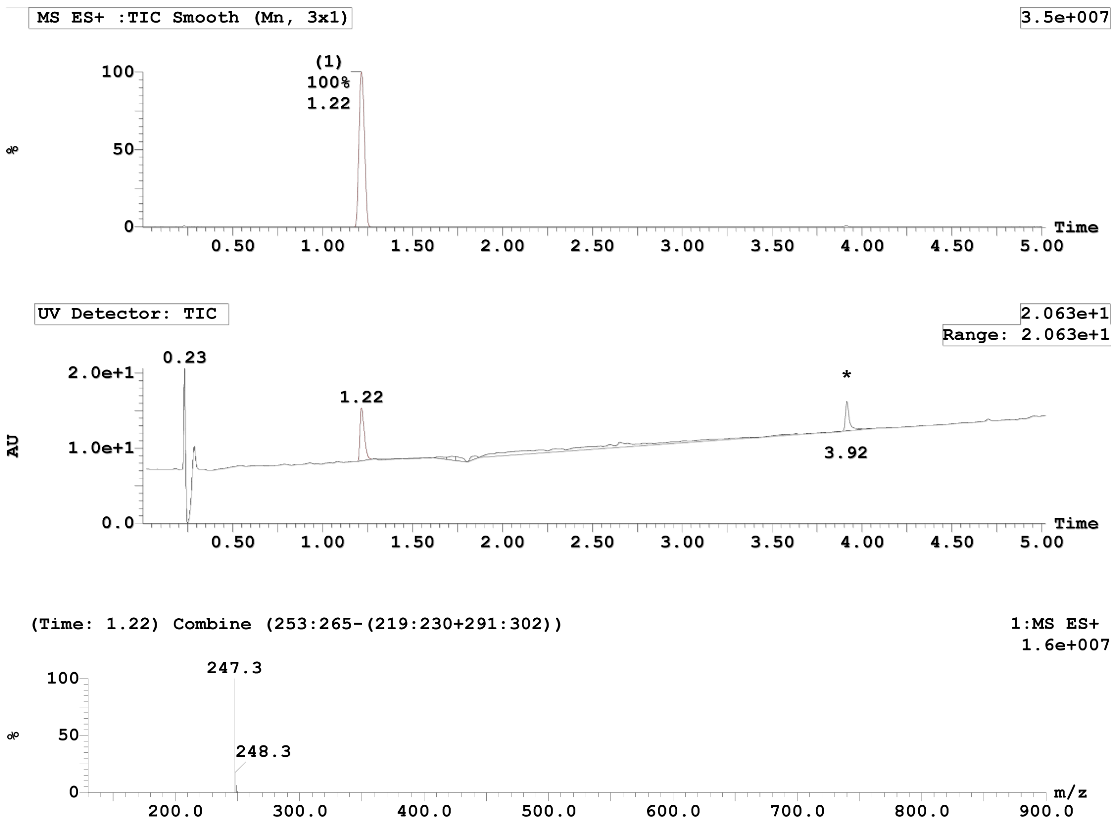
MS ES+ :TIC Smooth (Mn, 3x1)
3.5e+007
(1)
100%
1.22
%
Time
UV Detector: TIC
2.063e+1
Range: 2.063e+1
0.23
1.22
*
3.92
AU
Time
(Time: 1.22) Combine (253:265-(219:230+291:302))
1:MS ES+
1.6e+007
247.3
248.3
%
m/z

Compound **10**

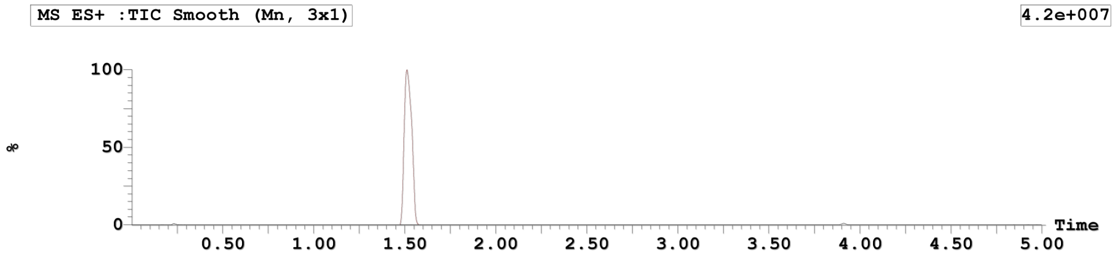
MS ES+ :TIC Smooth (Mn, 3x1)
4.2e+007
%
100
50
0
0.50
1.00
1.50
2.00
2.50
3.00
3.50
4.00
4.50
5.00
Time

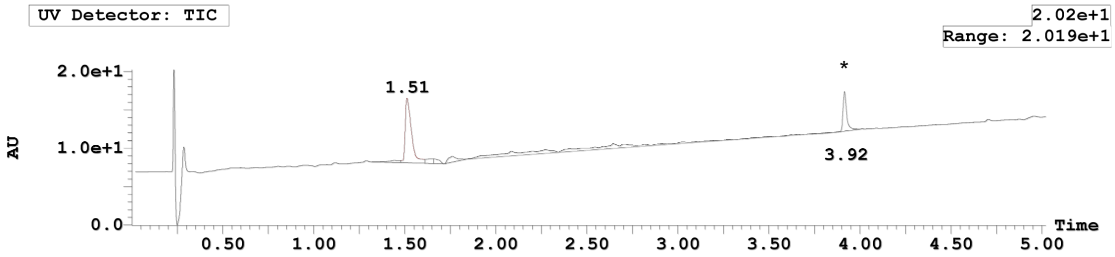
UV Detector: TIC
2.02e+1
Range: 2.019e+1
AU
2.0e+1
1.0e+1
0.0
1.51
*
3.92
0.50
1.00
1.50
2.00
2.50
3.00
3.50
4.00
4.50
5.00
Time

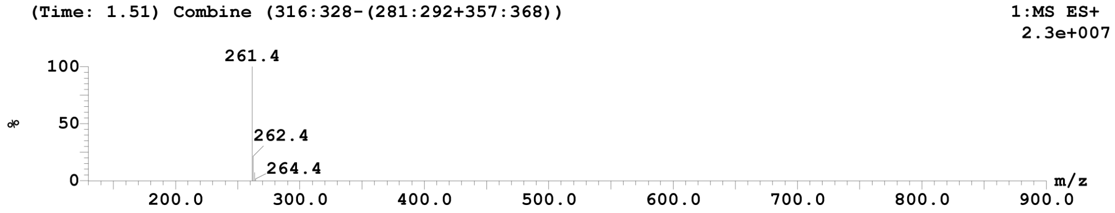
(Time: 1.51) Combine (316:328-(281:292+357:368))
1:MS ES+
2.3e+007
%
100
50
0
261.4
262.4
264.4
200.0
300.0
400.0
500.0
600.0
700.0
800.0
900.0
m/z

Compound **11a**

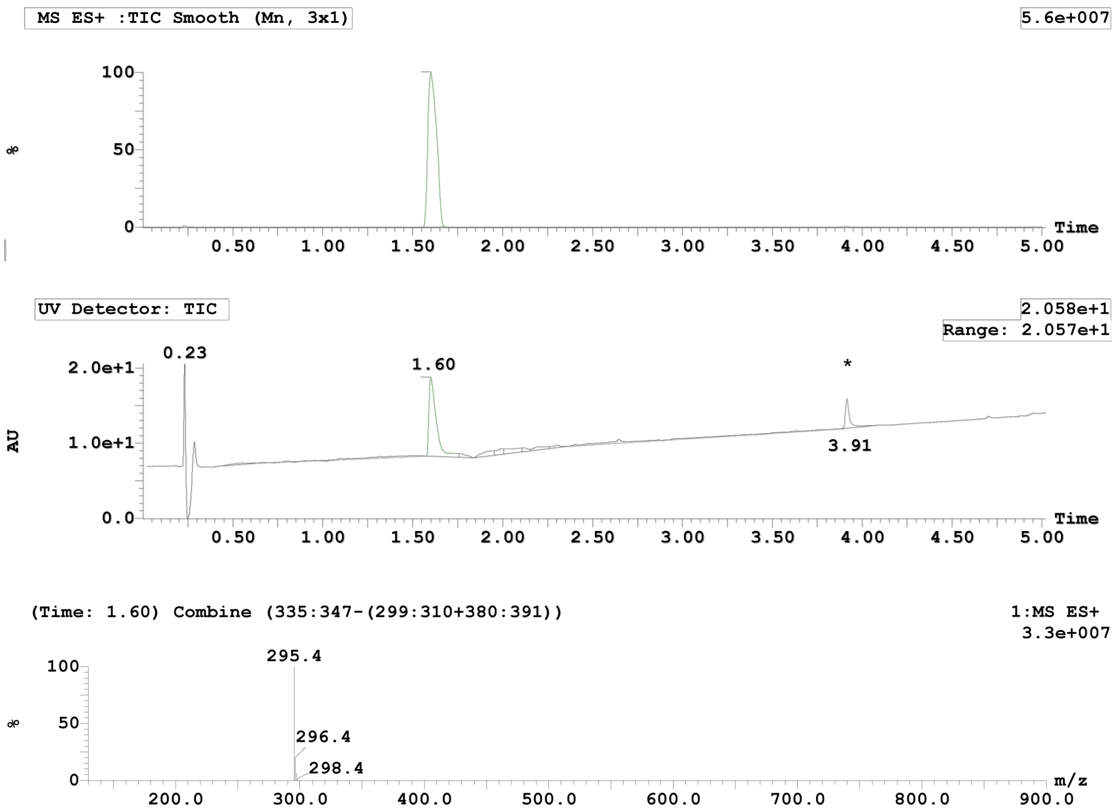

Compound **11b**

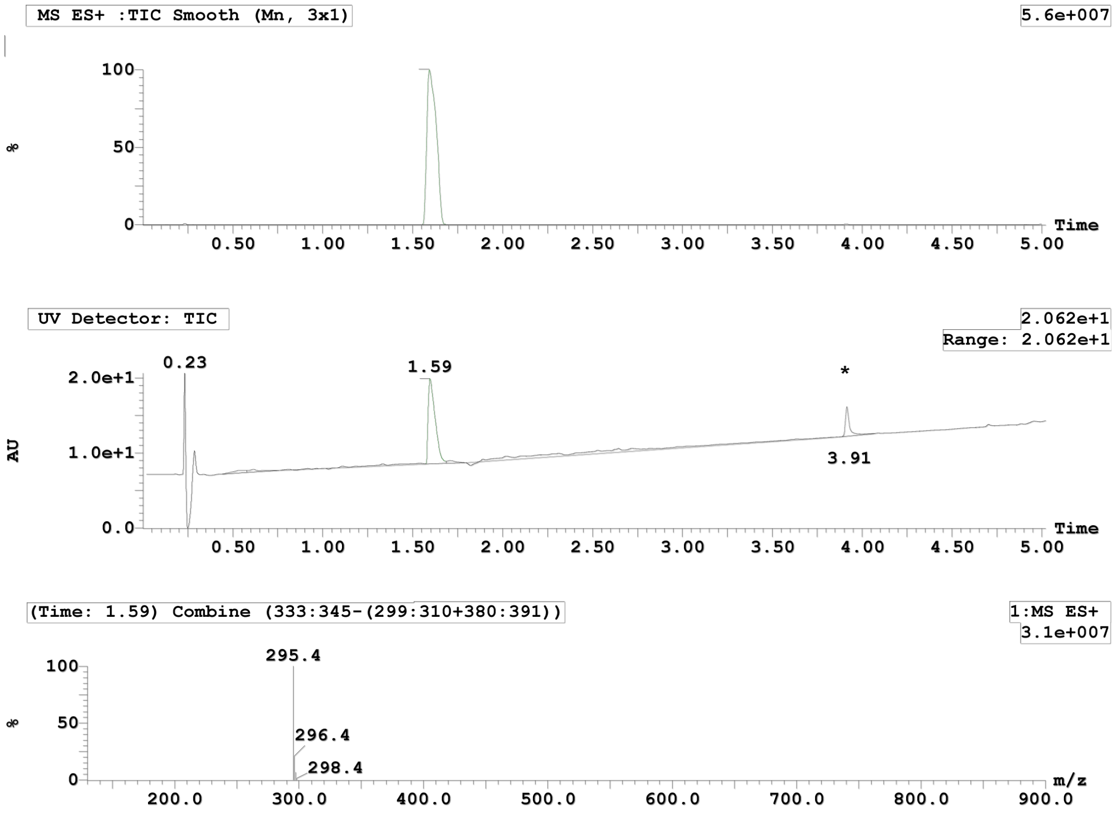

Compound **12**

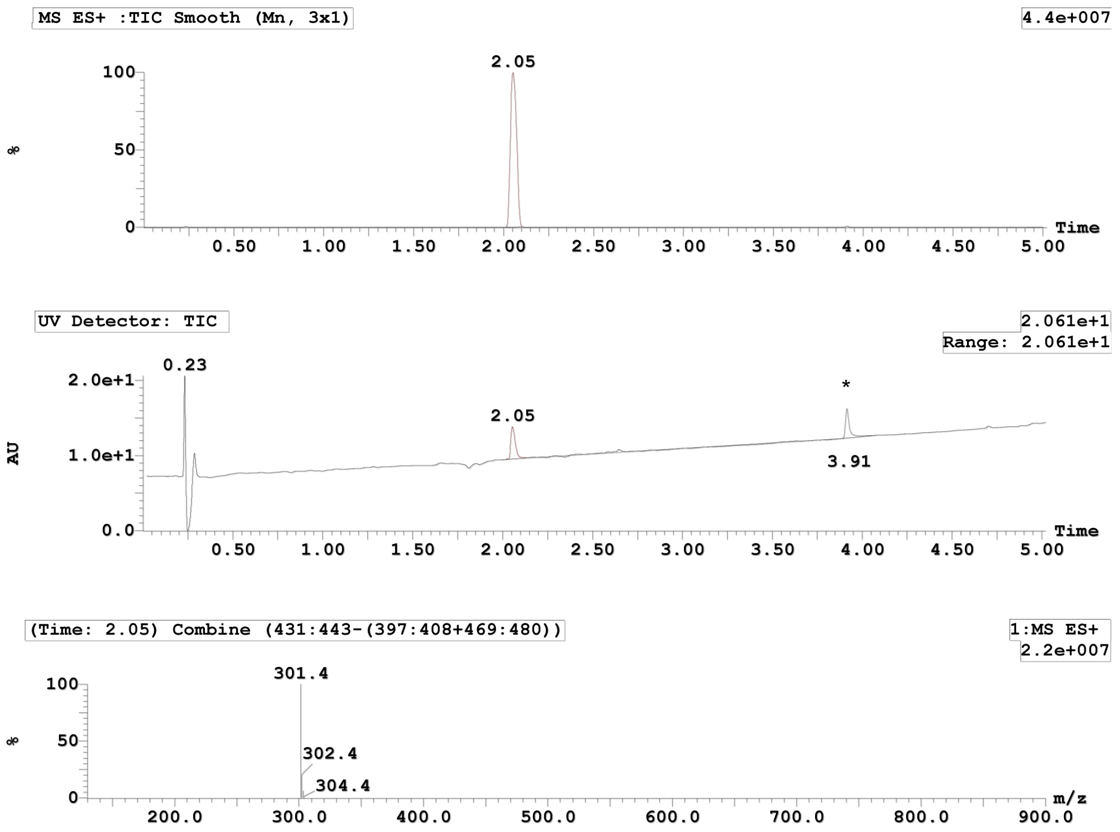
MS ES+ :TIC Smooth (Mn, 3x1)
4.4e+007
2.05
Time
UV Detector: TIC
2.061e+1
Range: 2.061e+1
0.23
2.05
*
3.91
AU
(Time: 2.05) Combine (431:443-(397:408+469:480))
1:MS ES+
2.2e+007
301.4
302.4
304.4
m/z

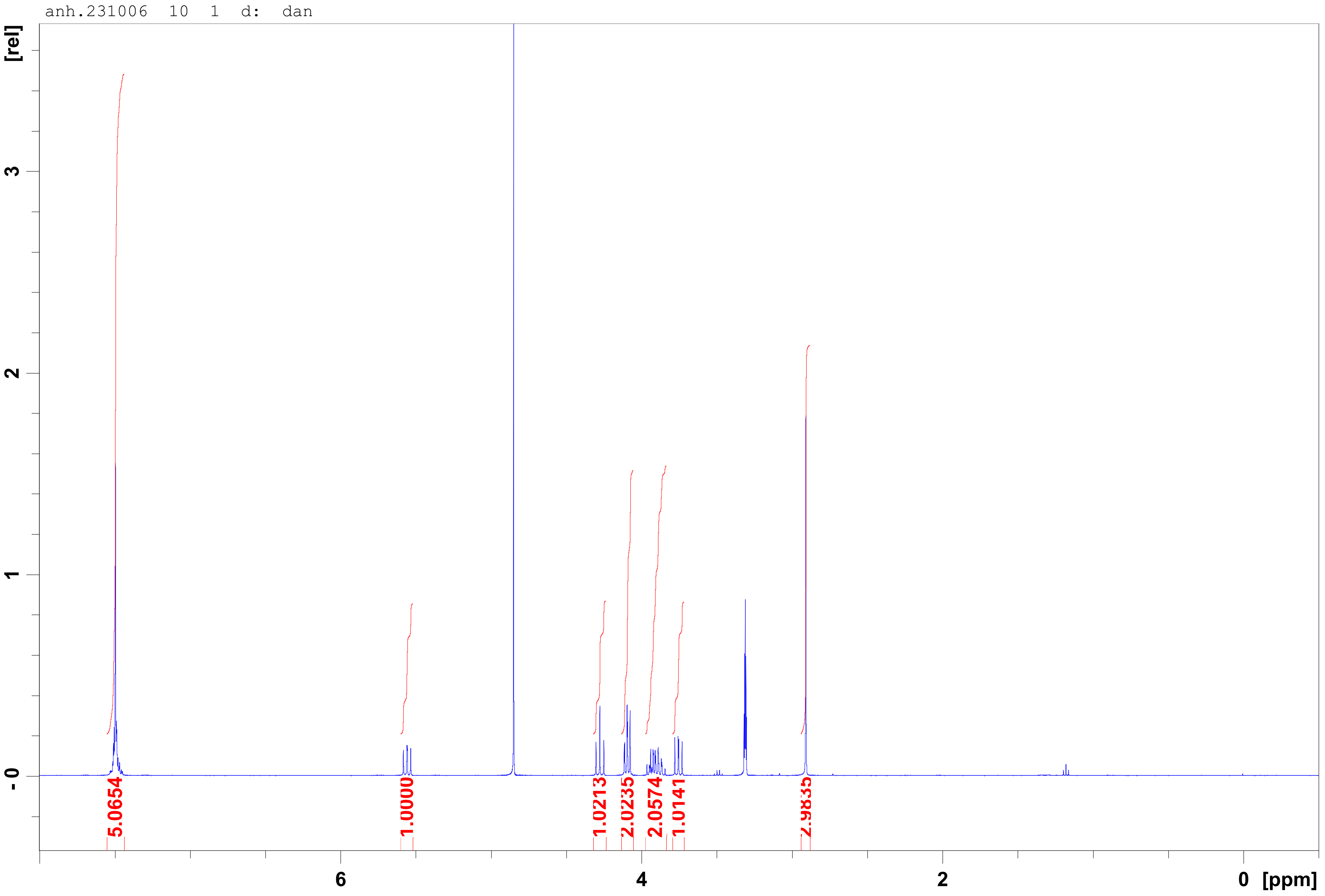
anh.231006 10 1 d: dan
[rel]
3
2
1
- 0
5.0654
1.0000
1.0213
2.0235
2.0574
1.0141
2.9835
6
4
2
0 [ppm]

anh.231006 11 1 d: dan

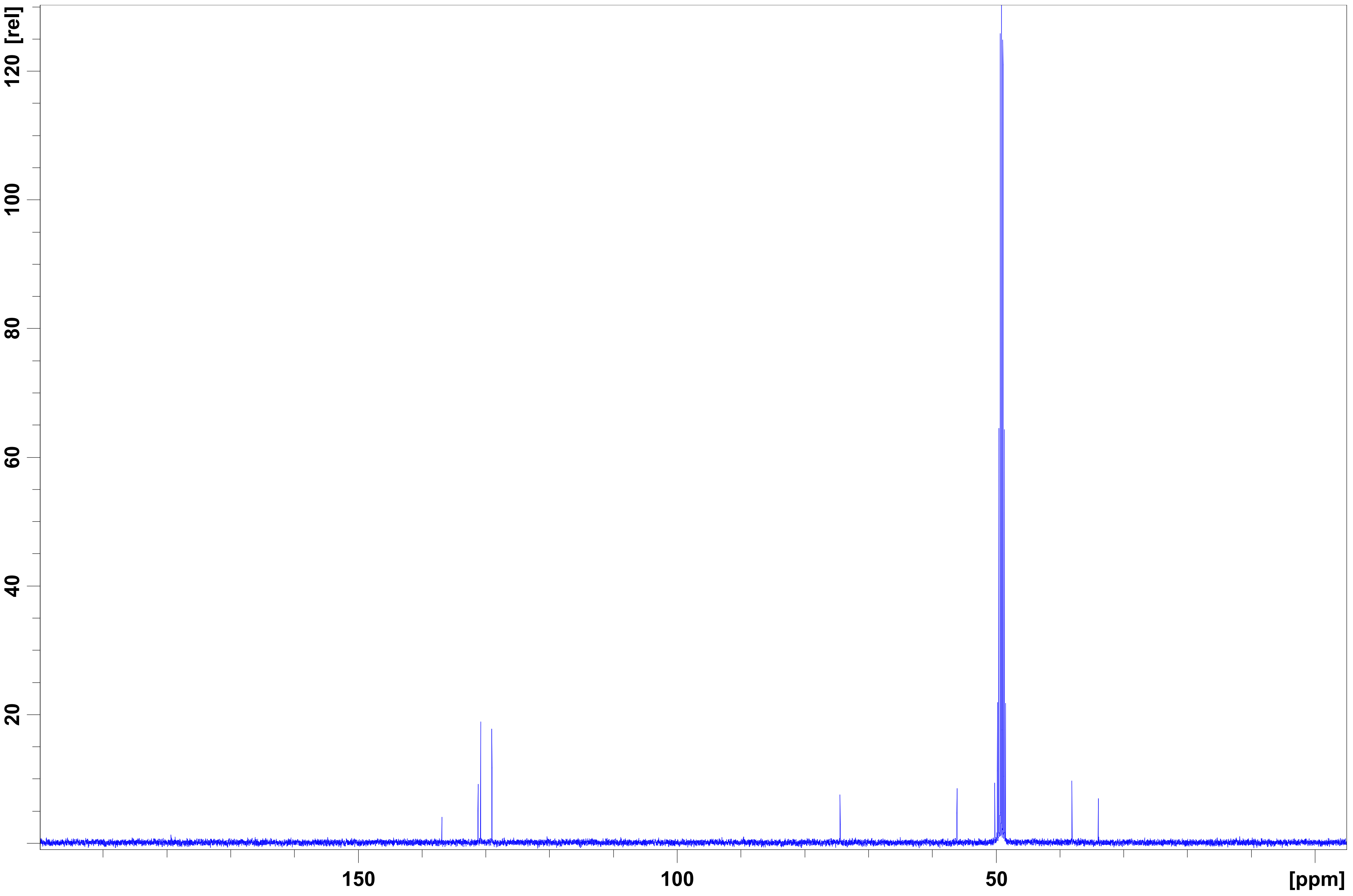

anh.231006 12 1 d: dan

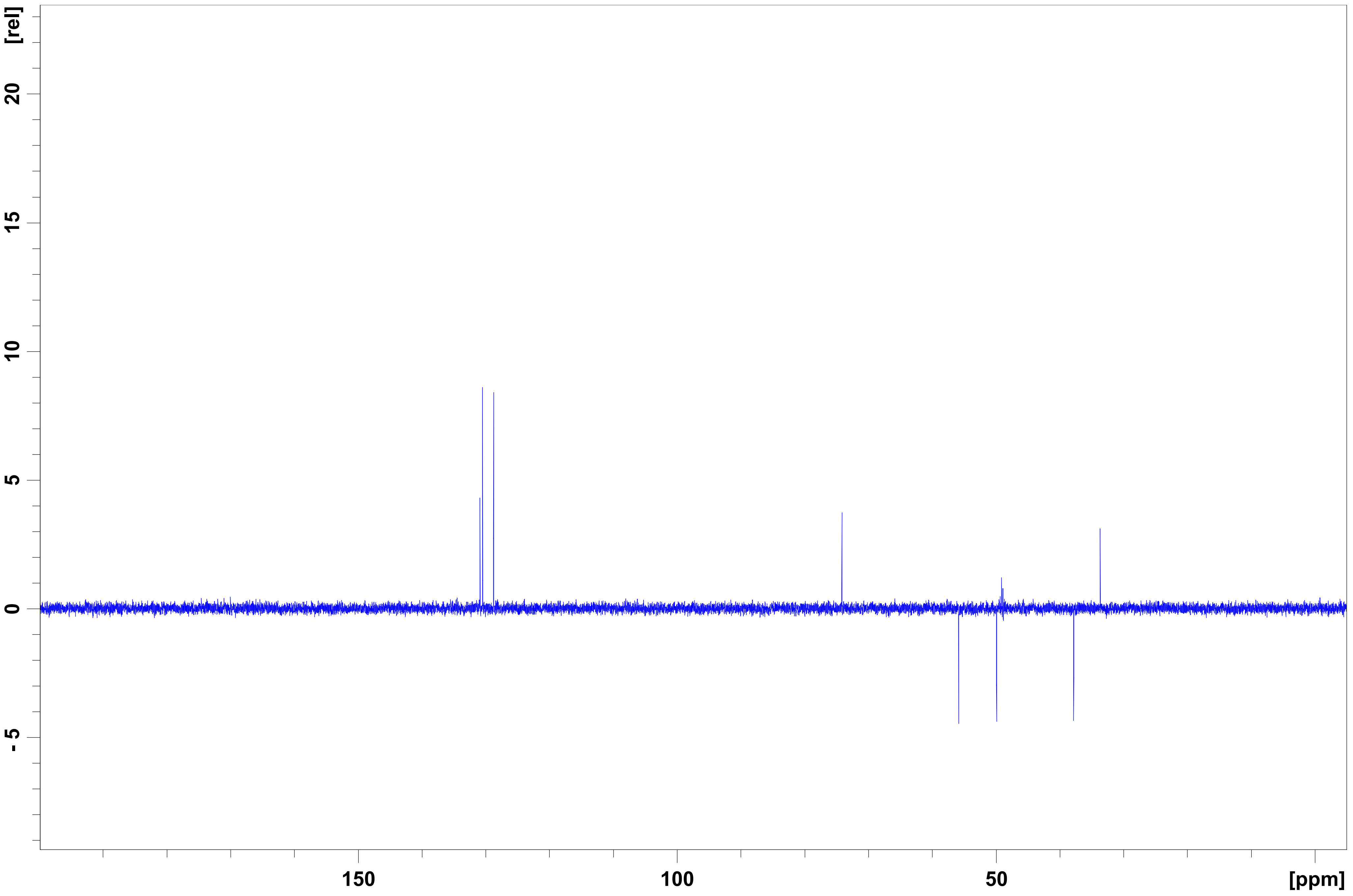

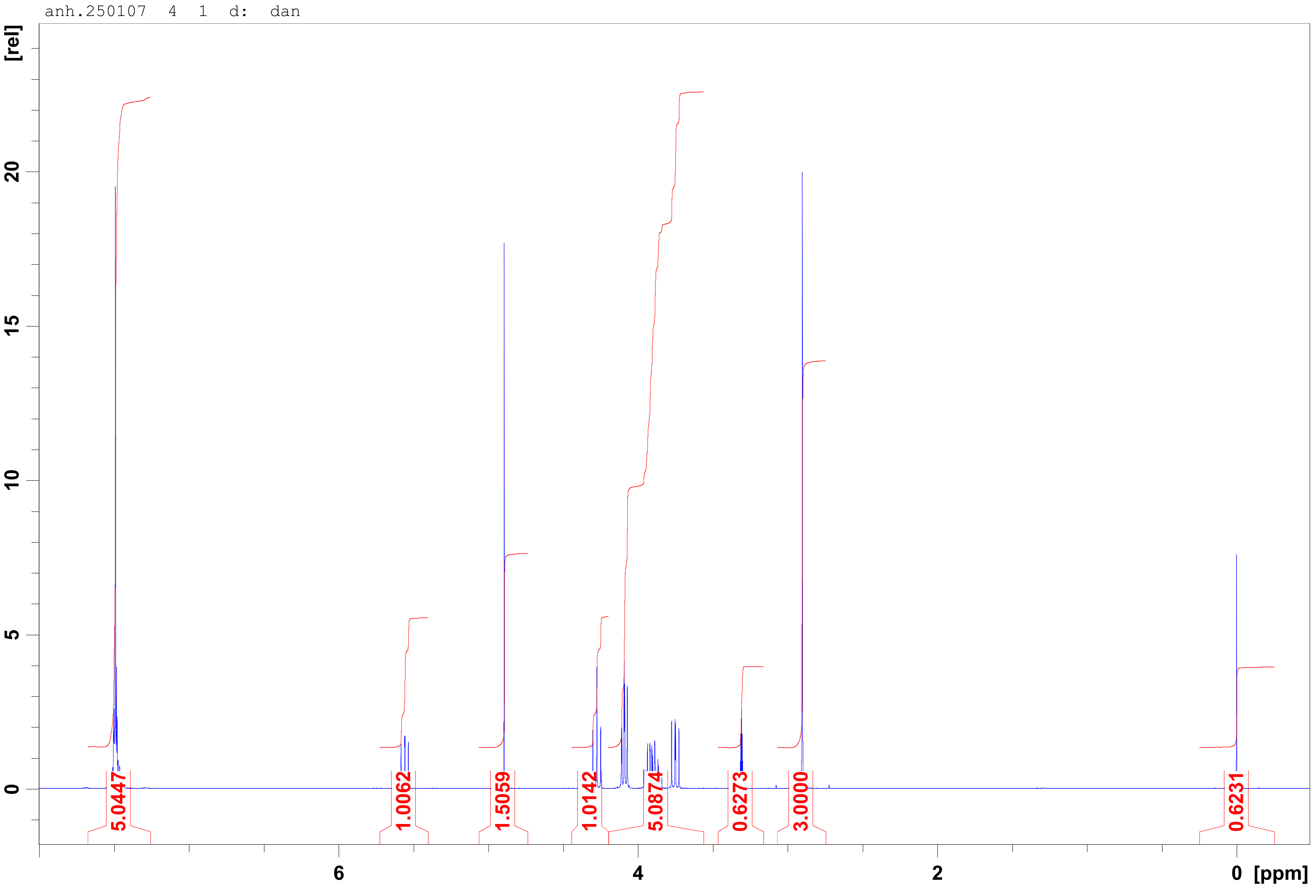
anh.250107 4 1 d: dan
[rel]
20
15
10
5
0
5.0447
1.0062
1.5059
1.0142
5.0874
0.6273
3.0000
0.6231
6
4
2
0 [ppm]

anh.250107 5 1 d: dan

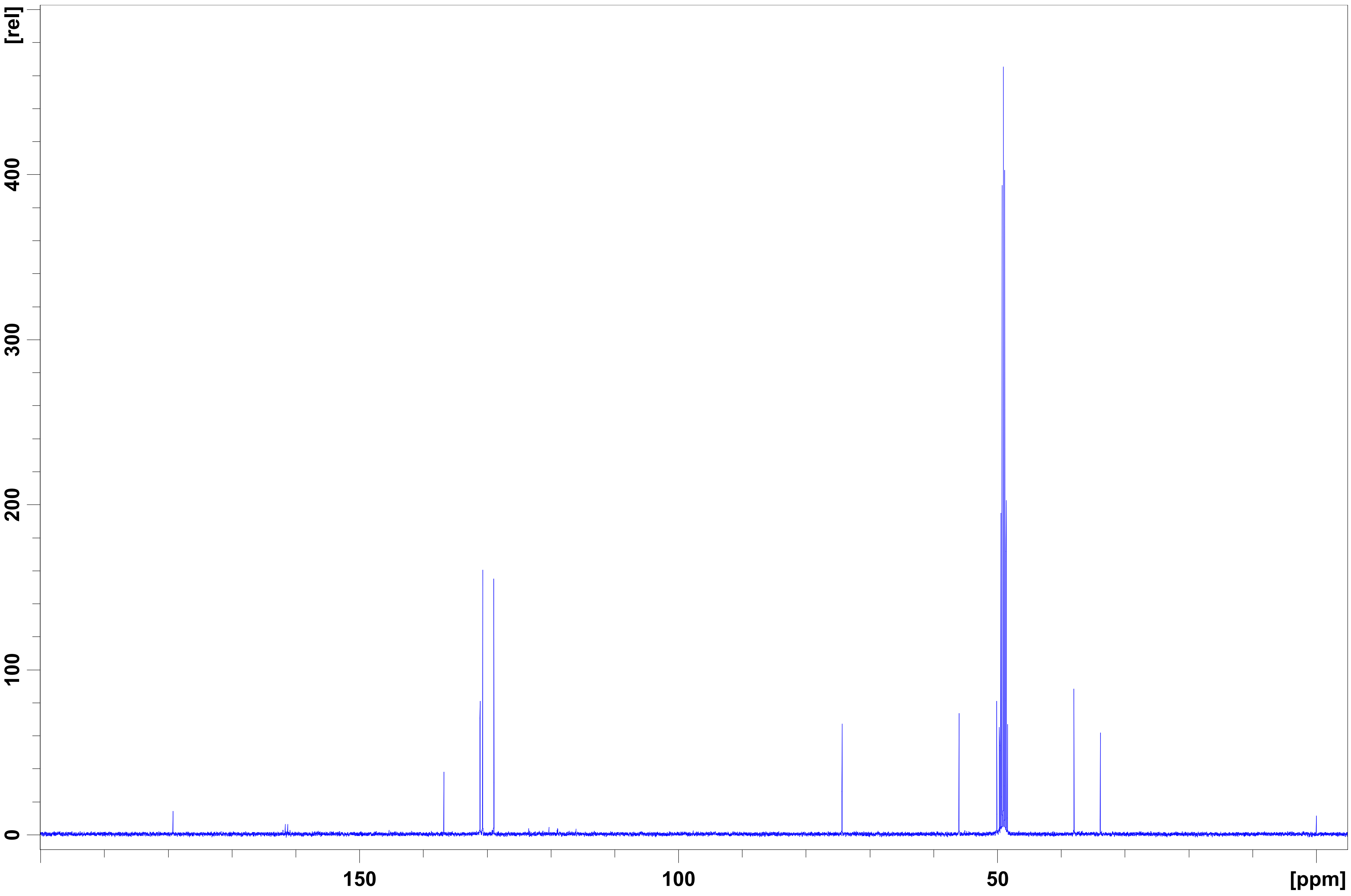

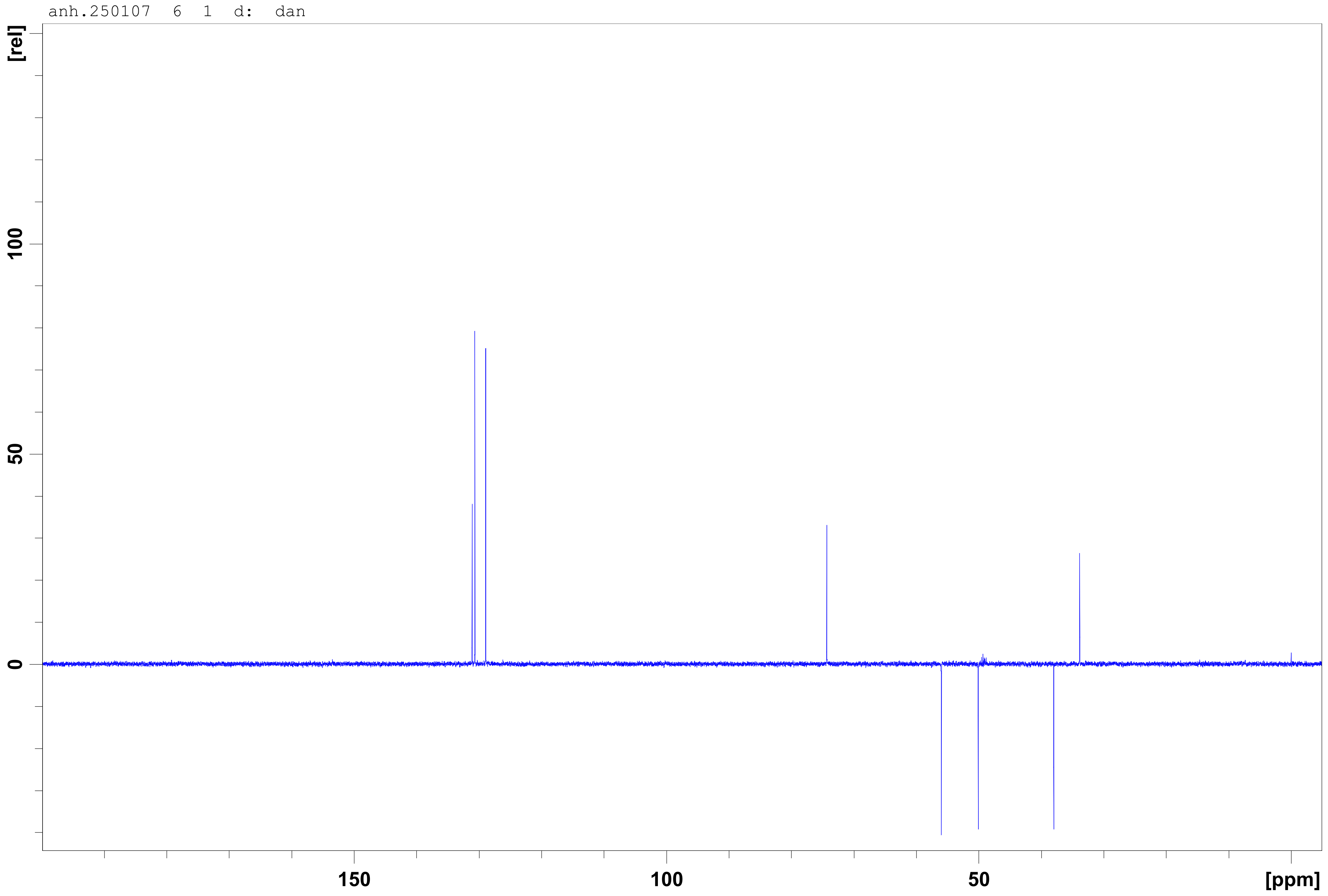
anh.250107 6 1 d: dan
[rel]
100
50
0
150
100
50
[ppm]

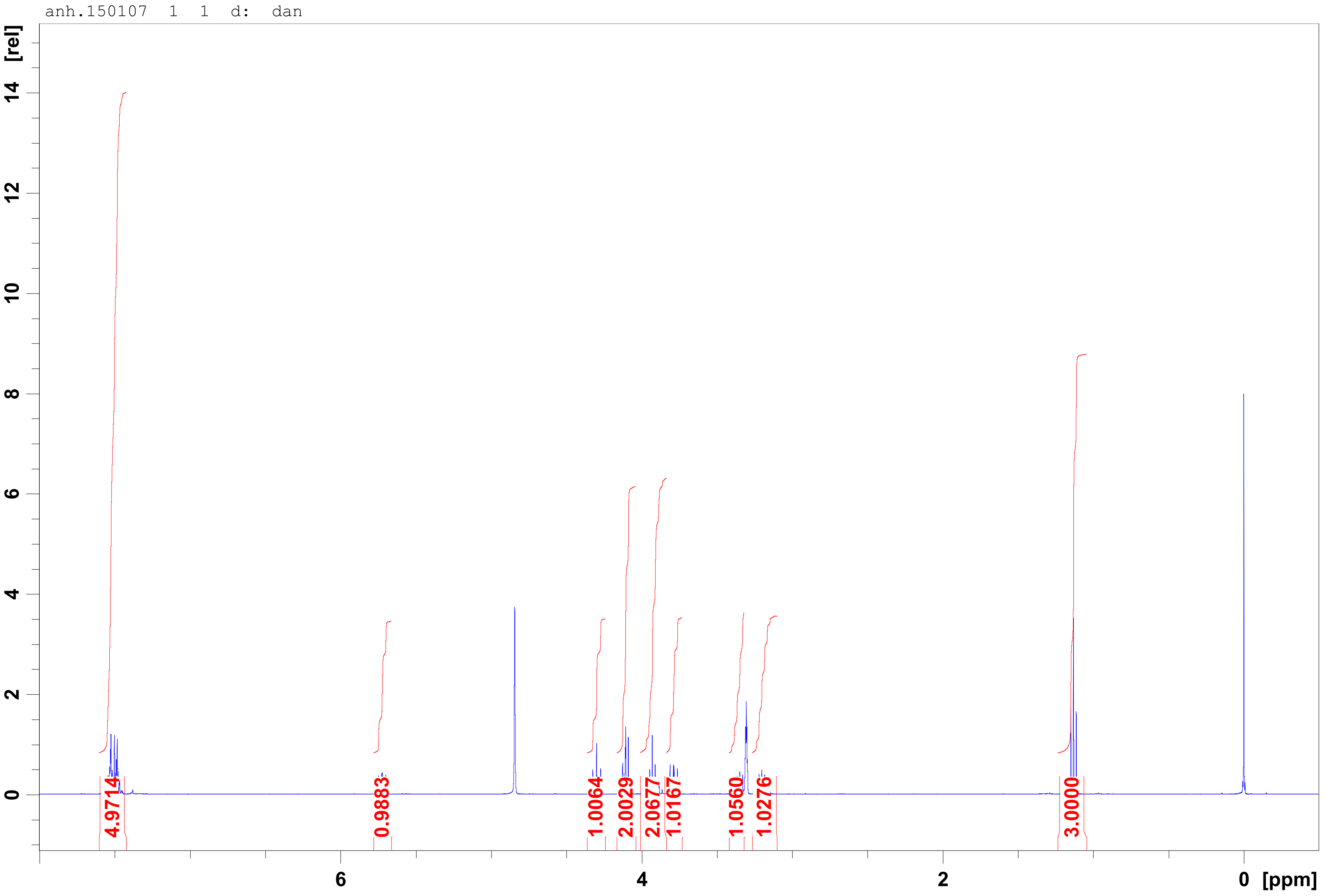
anh.150107 1 1 d: dan
[rel]
14
12
10
8
6
4
2
0
4.9714
0.9883
1.0064
2.0029
2.0677
1.0167
1.0560
1.0276
3.0000
6
4
2
0 [ppm]

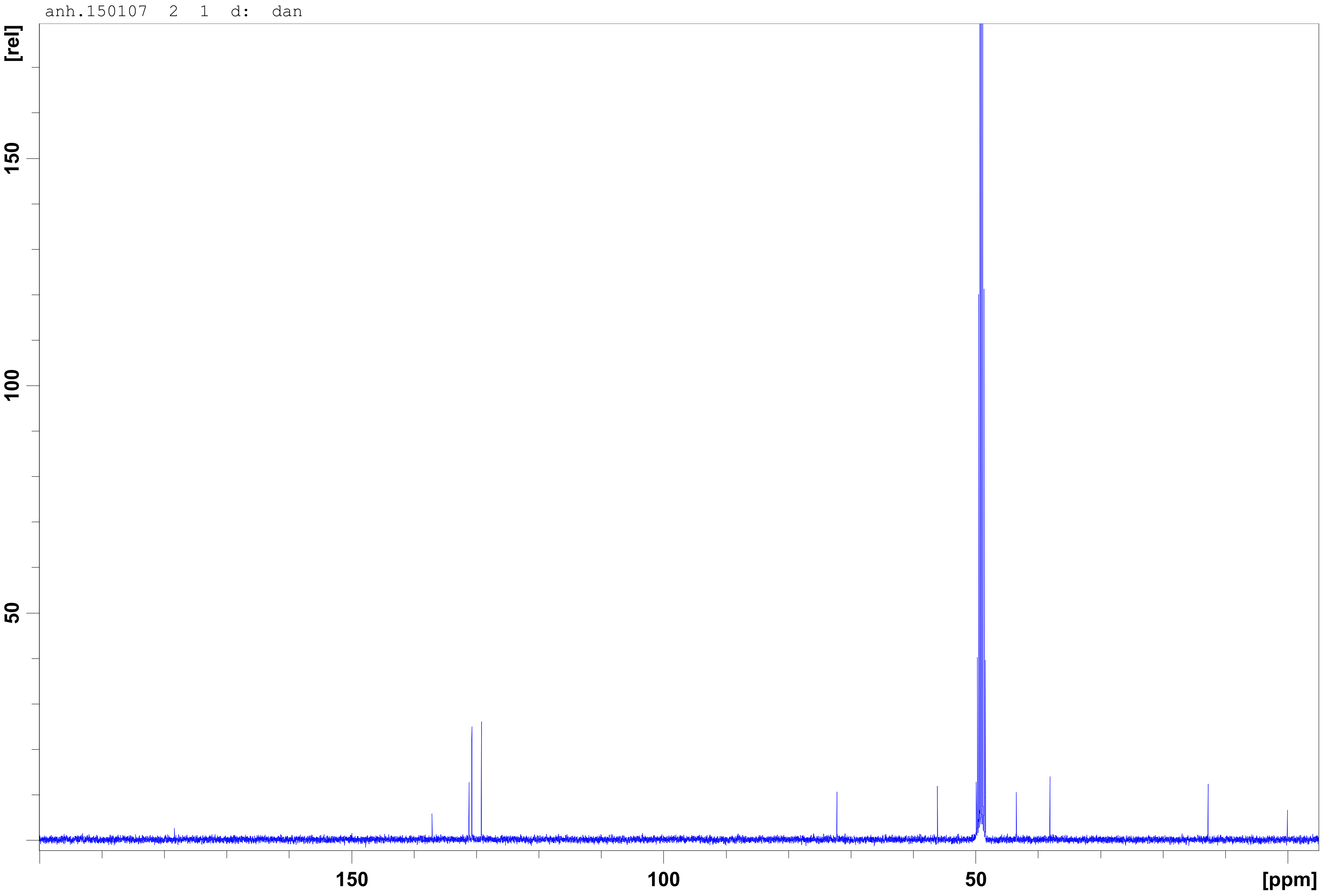
anh.150107 2 1 d: dan
[rel]
150
100
50
150
100
50
[ppm]

anh.150107 3 1 d: dan

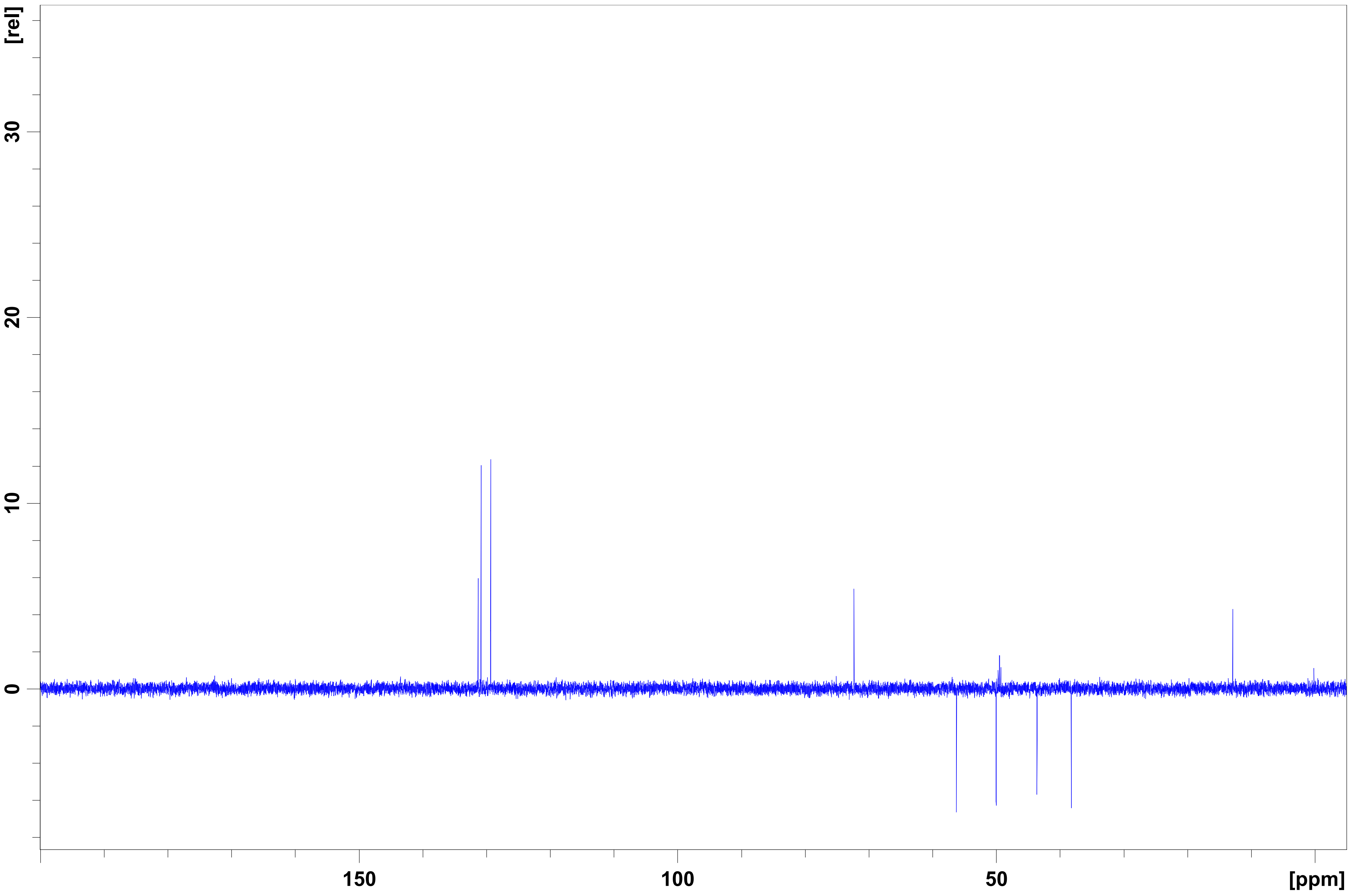

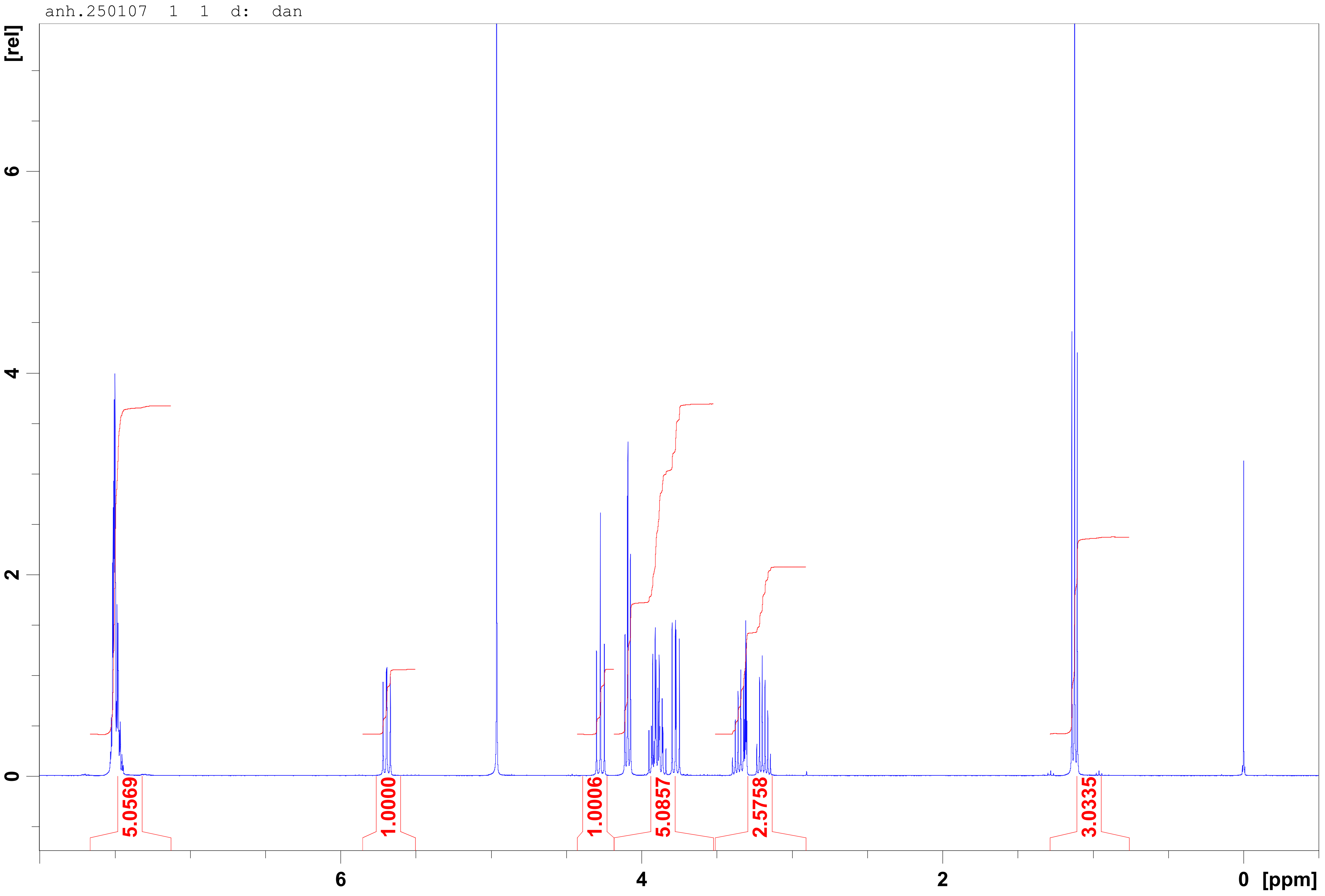
anh.250107 1 1 d: dan
[rel]
6
4
2
0
5.0569
1.0000
1.0006
5.0857
2.5758
3.0335
6
4
2
0 [ppm]

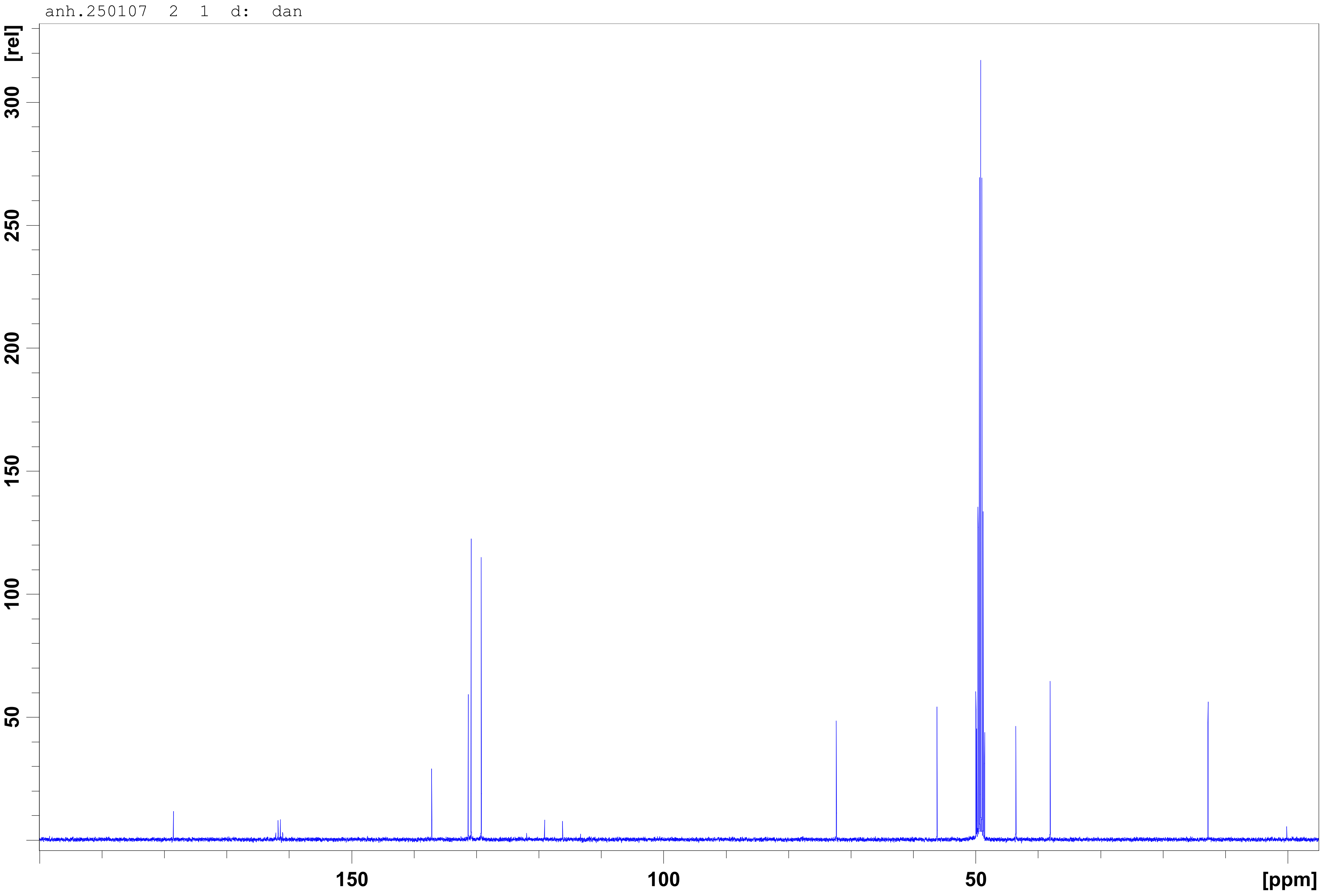
anh.250107 2 1 d: dan
[rel]
300
250
200
150
100
50
150
100
50
[ppm]

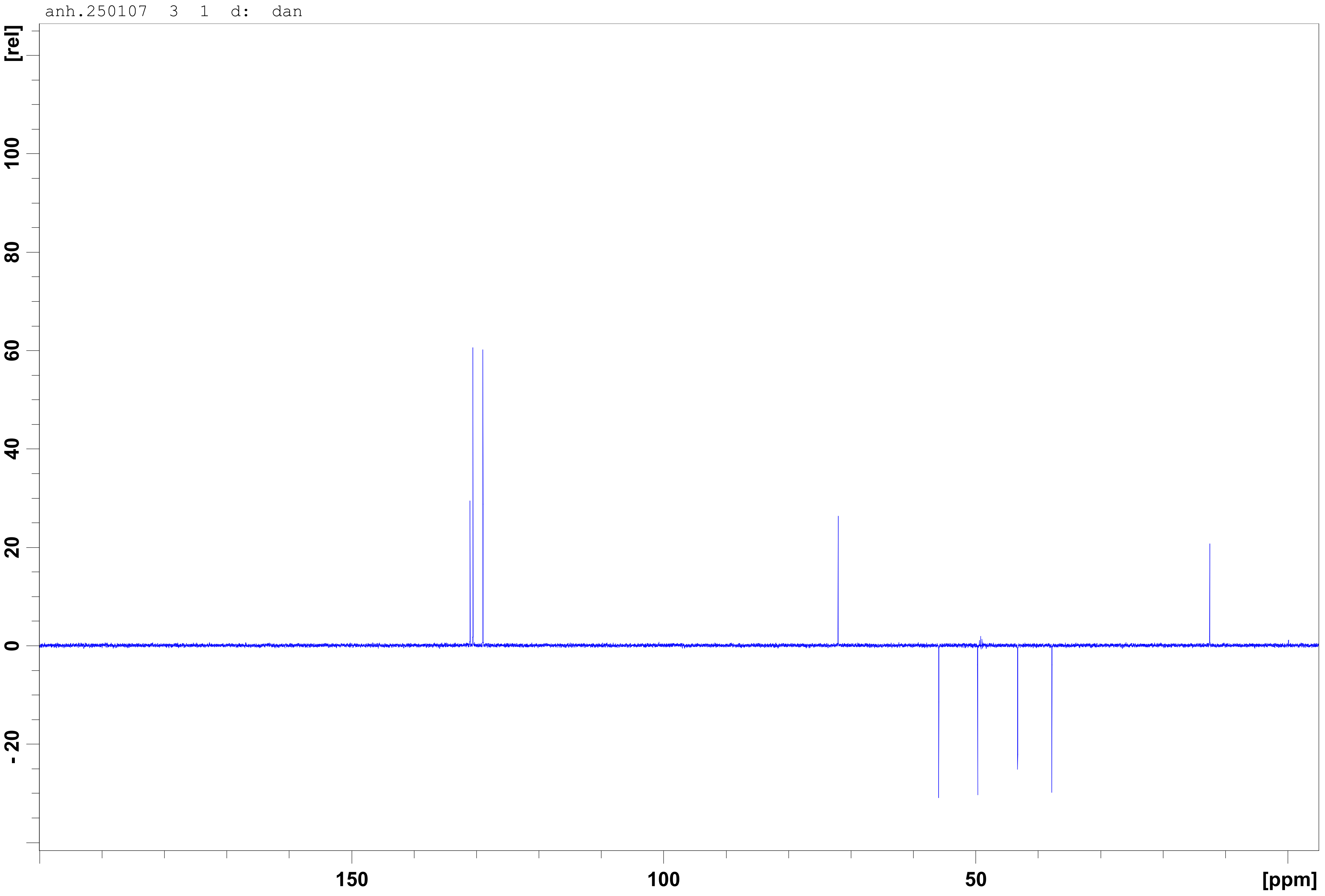
anh.250107 3 1 d: dan
[rel]
100
80
60
40
20
0
- 20
150
100
50
[ppm]

anh.240107 1 1 d: dan

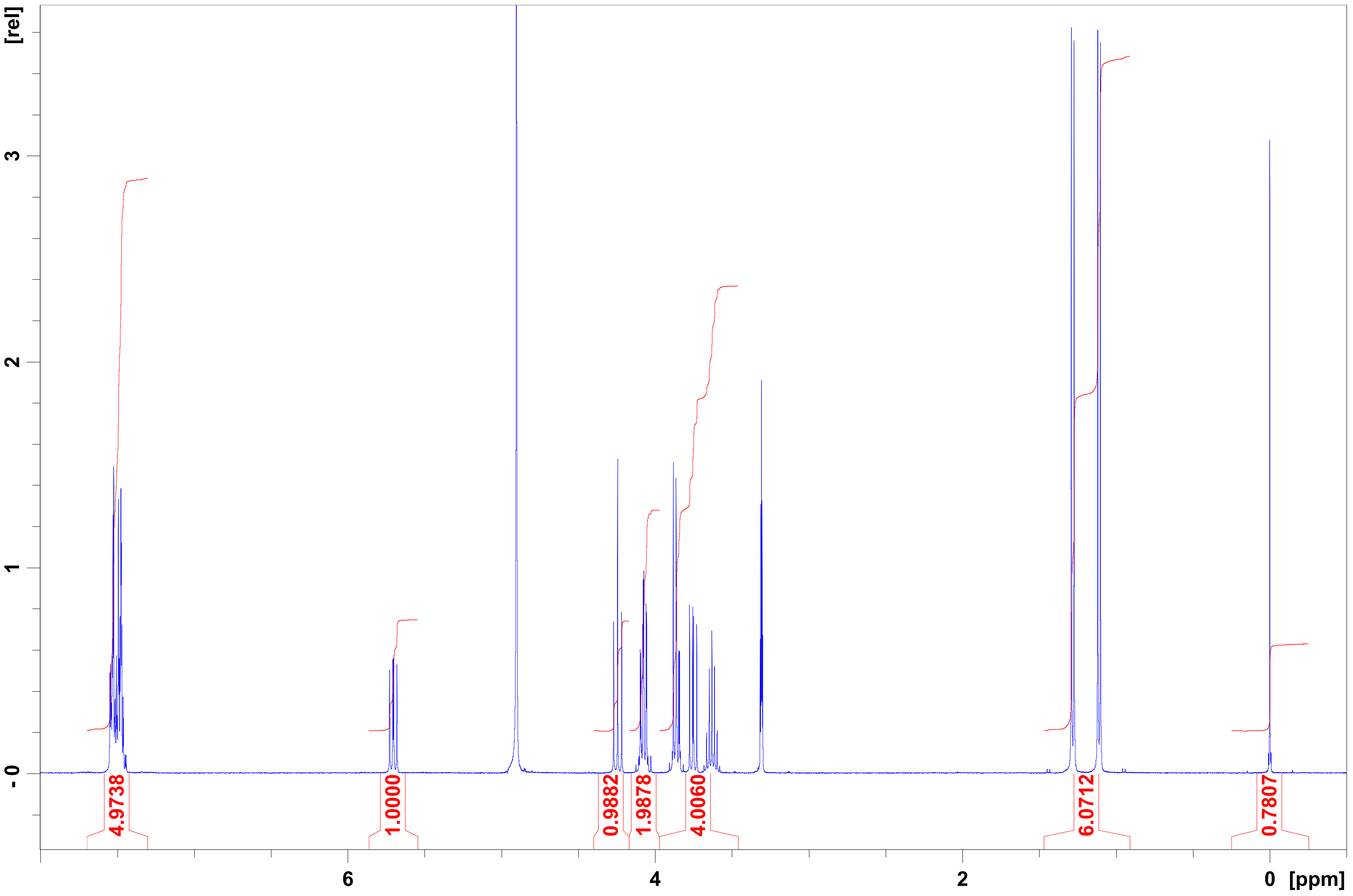

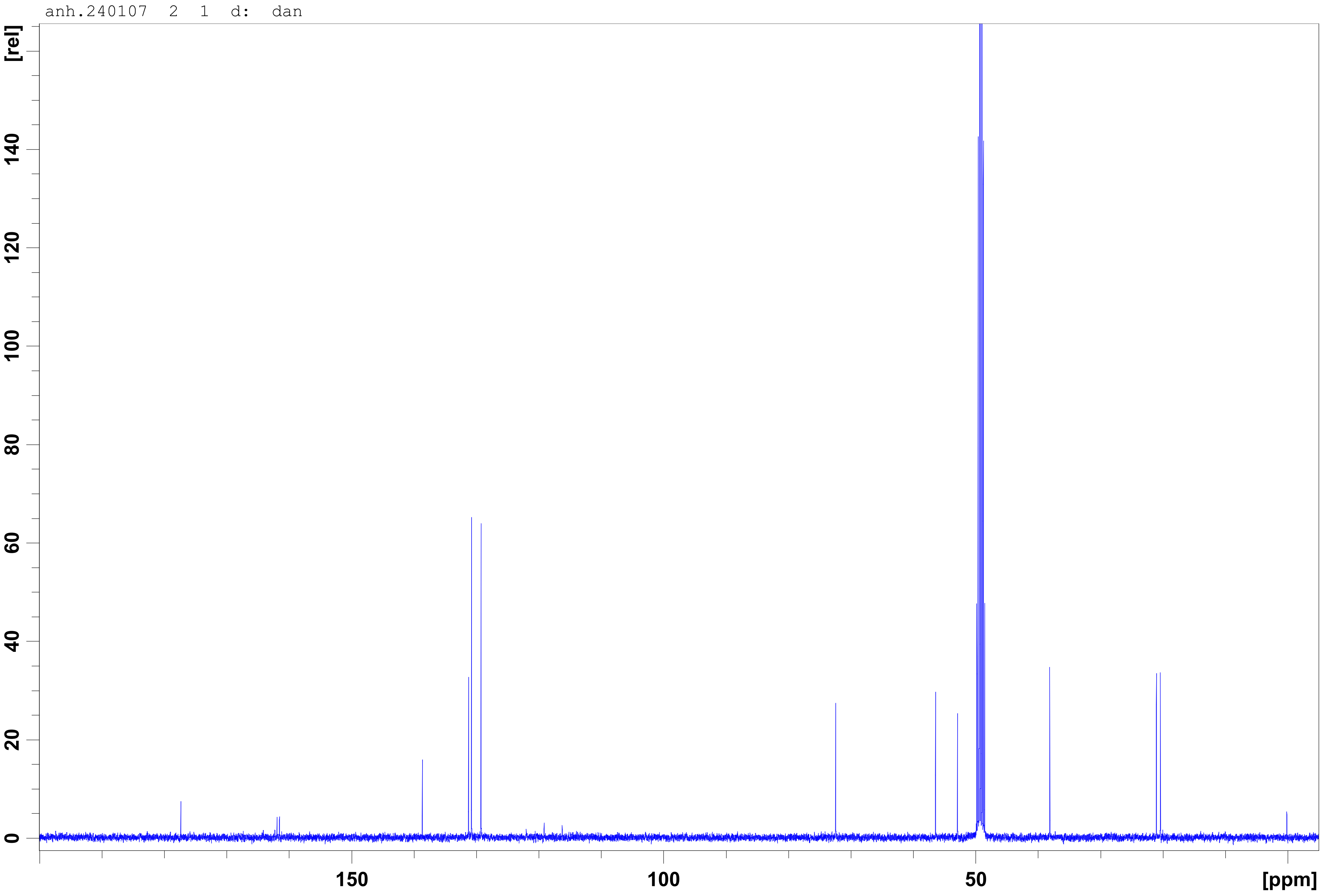

anh.240107 2 1 d: dan
[rel]
140
120
100
80
60
40
20
0
150
100
50
[ppm]

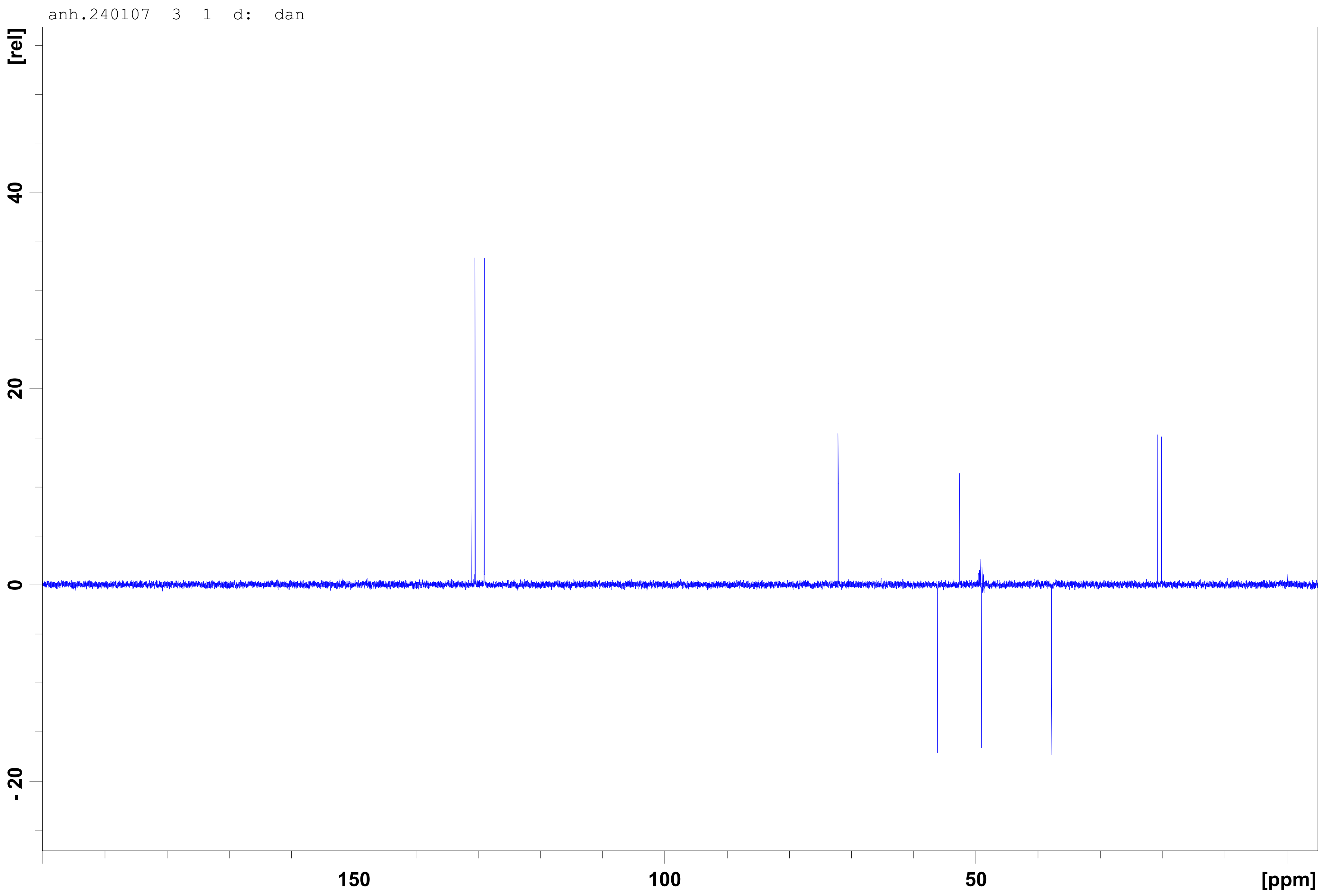
anh.240107 3 1 d: dan
[rel]
40
20
0
- 20
150
100
50
[ppm]

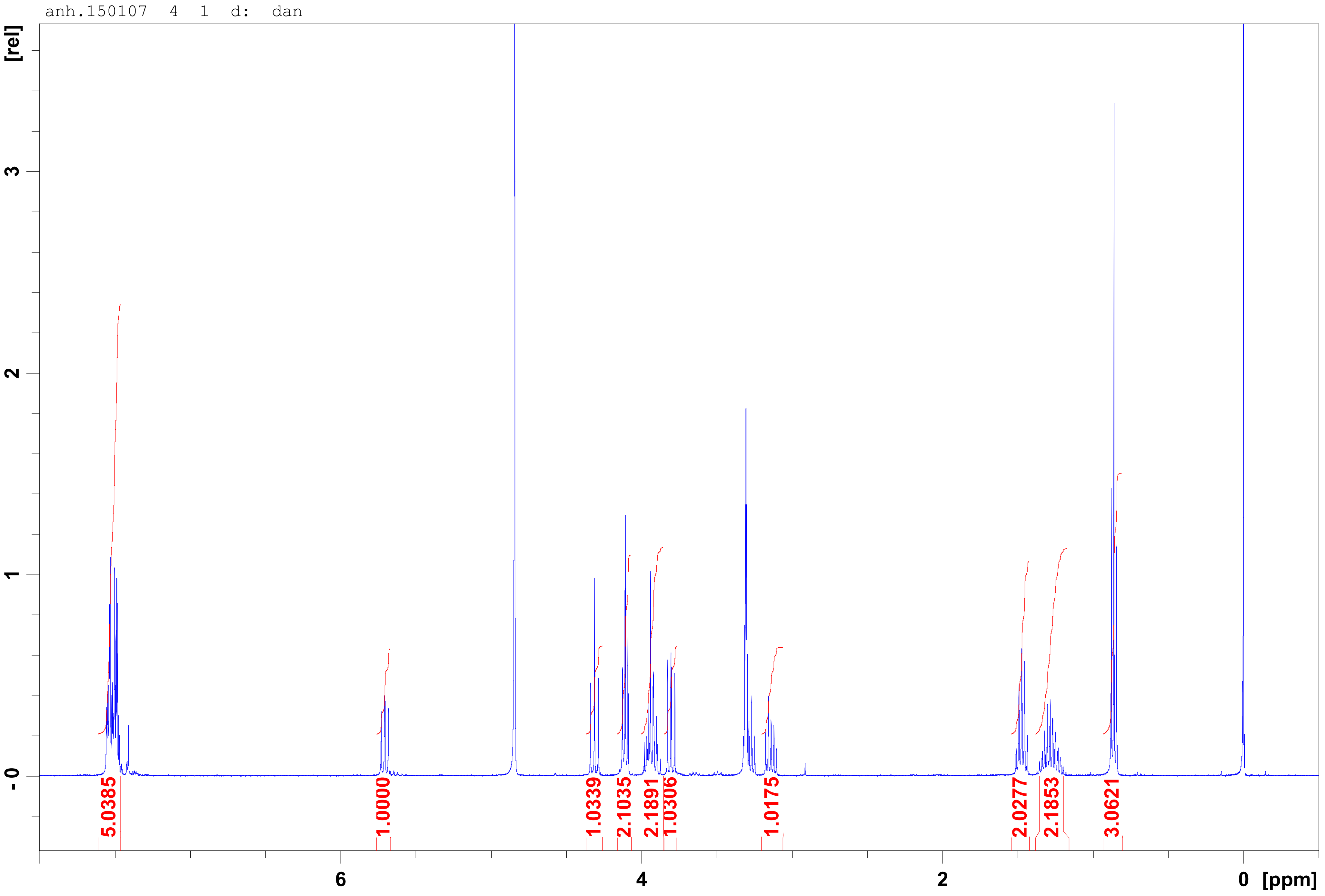
anh.150107 4 1 d: dan
[rel]
3
2
1
- 0
5.0385
1.0000
1.0339
2.1035
2.1891
1.0306
1.0175
2.0277
2.1853
3.0621
6
4
2
0 [ppm]

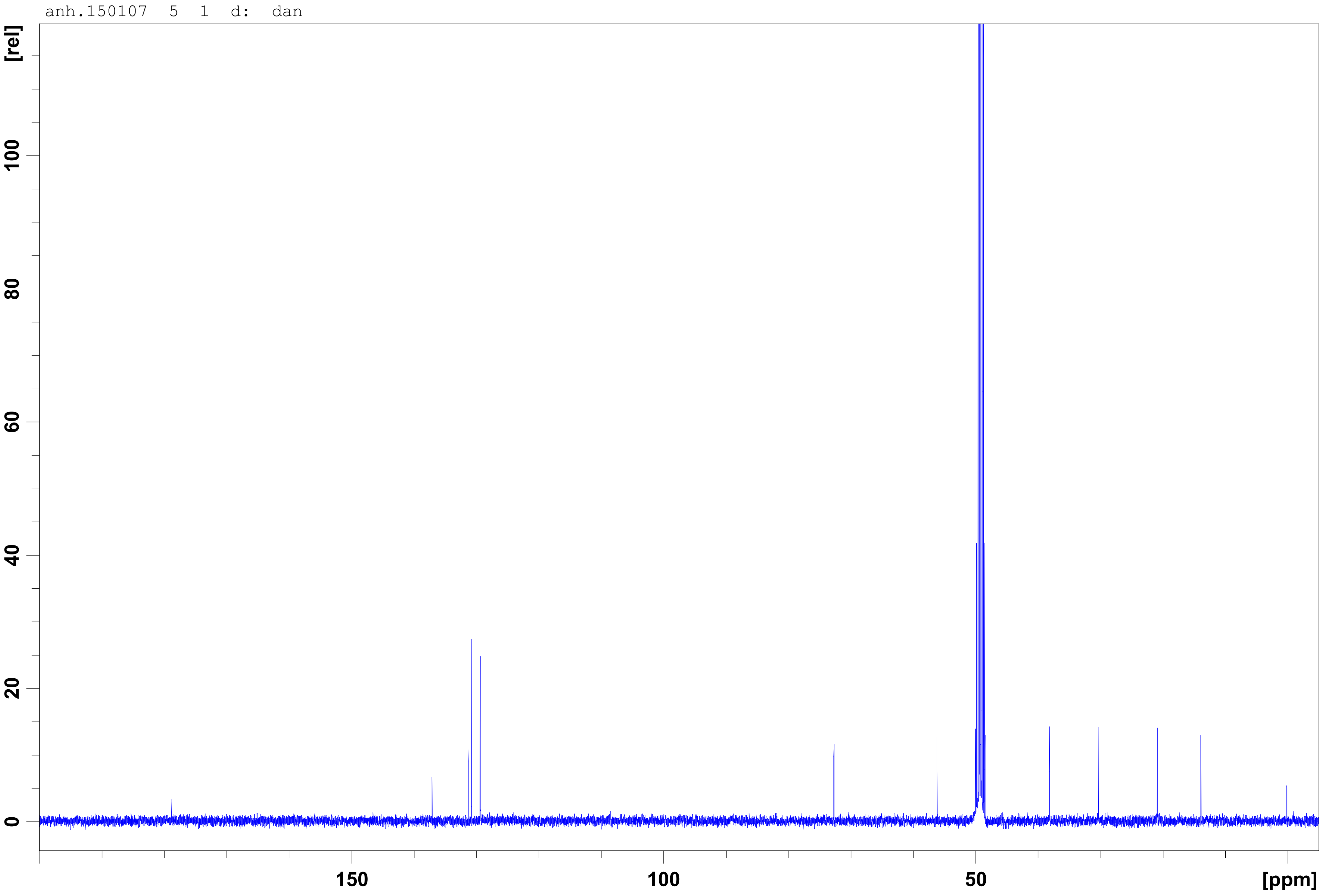
anh.150107 5 1 d: dan
[rel]
100
80
60
40
20
0
150
100
50
[ppm]

anh.150107 6 1 d: dan

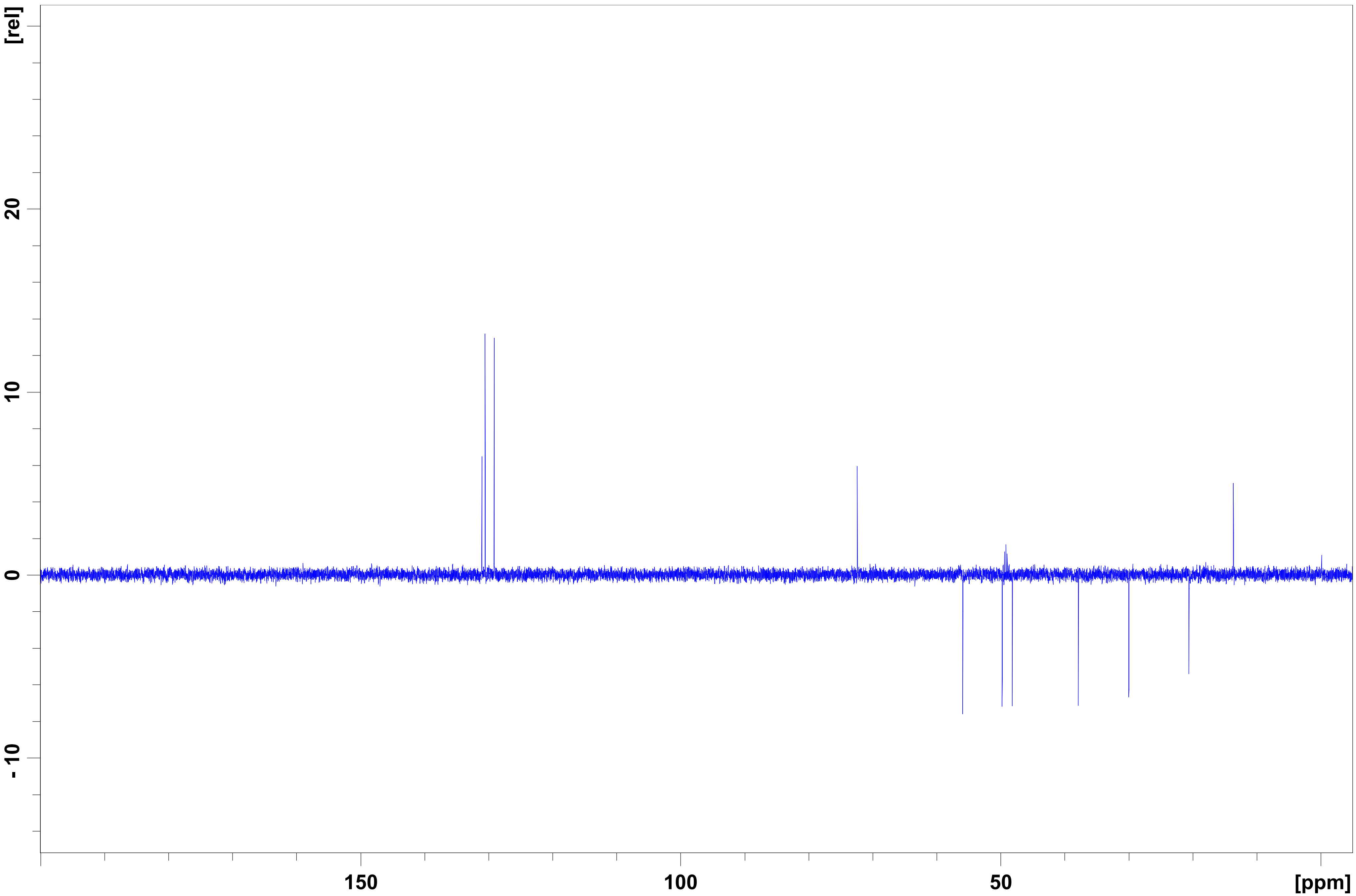

anh.160107 1 1 d: dan

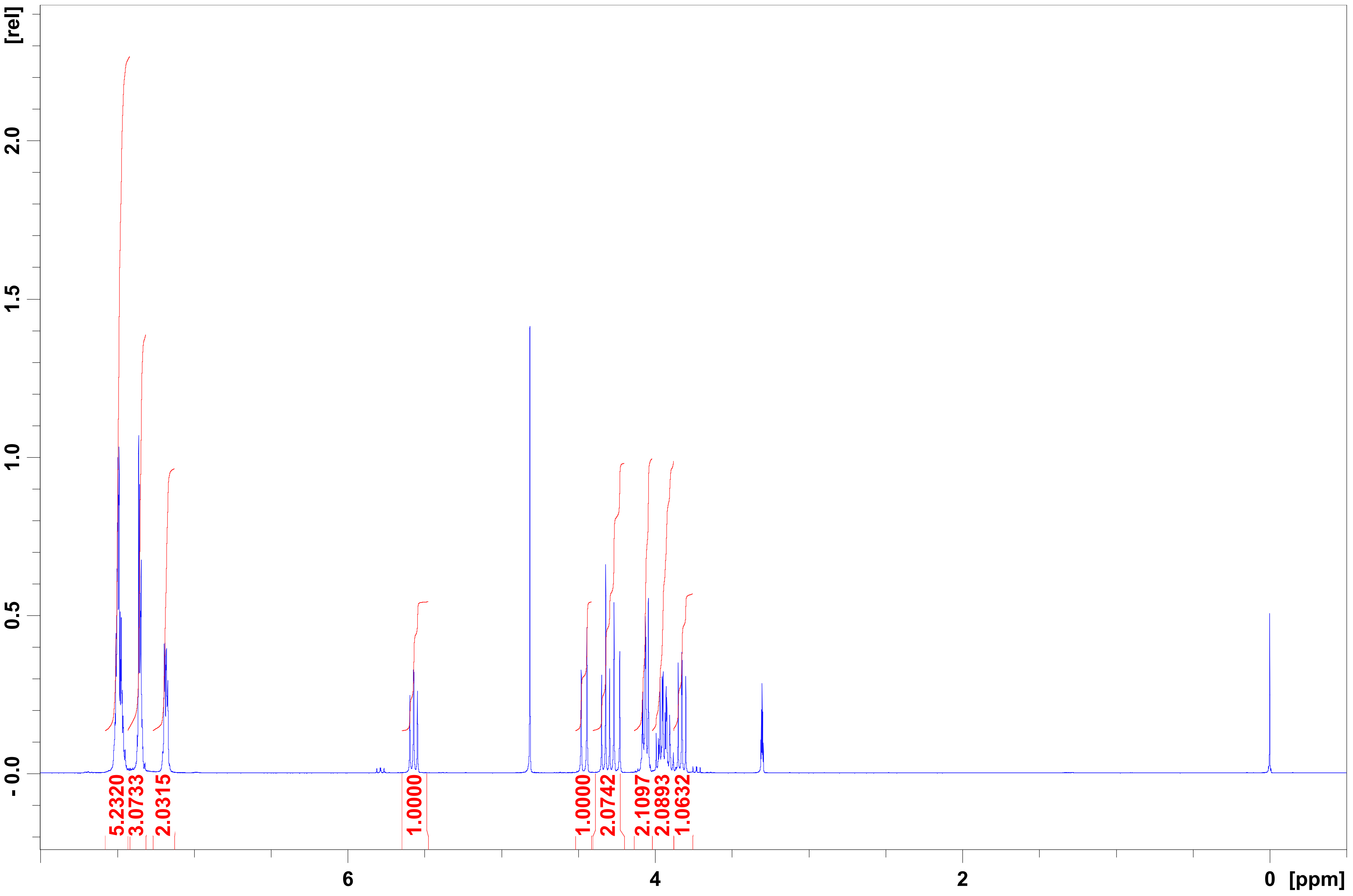

anh.160107 2 1 d: dan

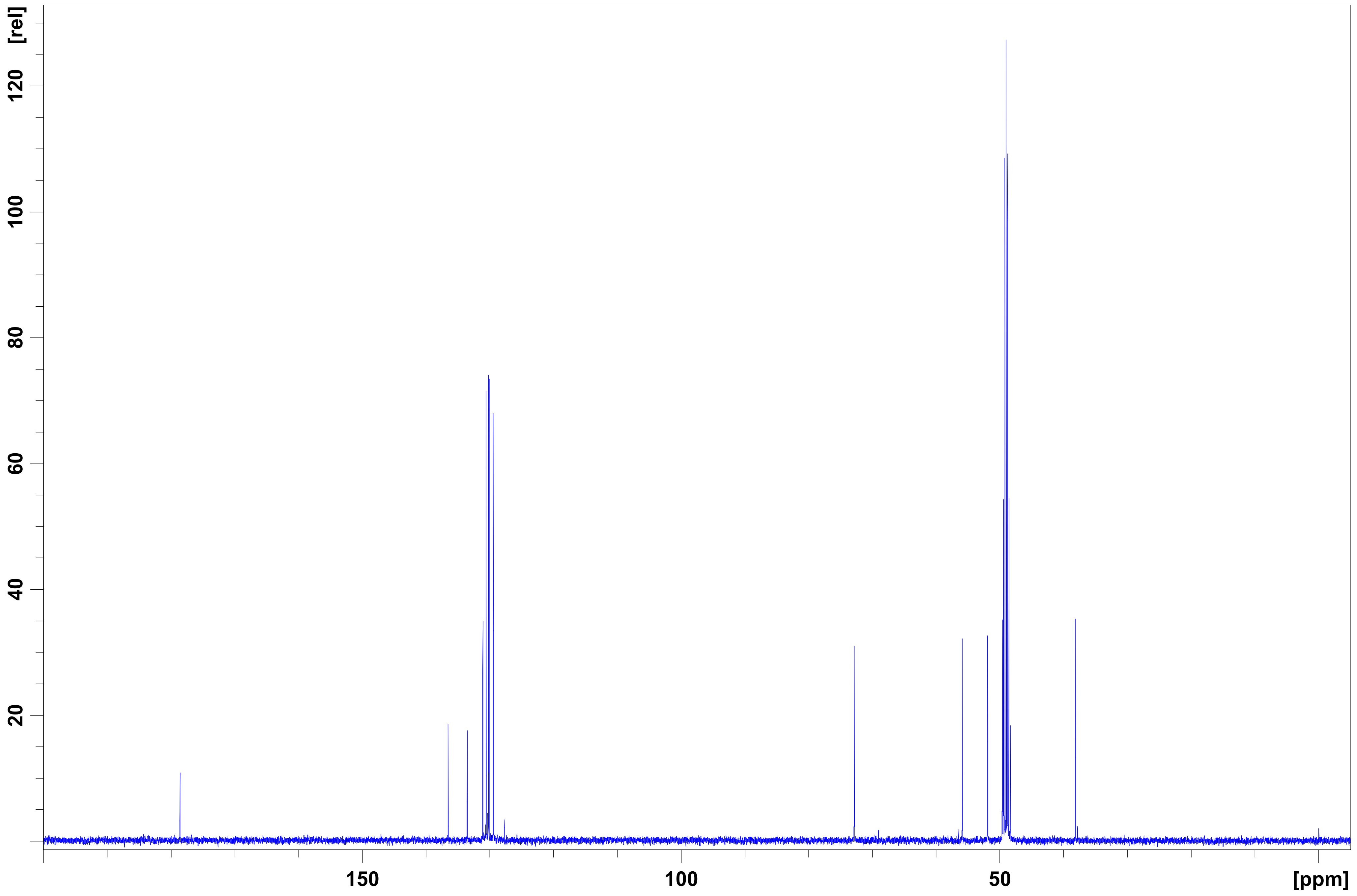

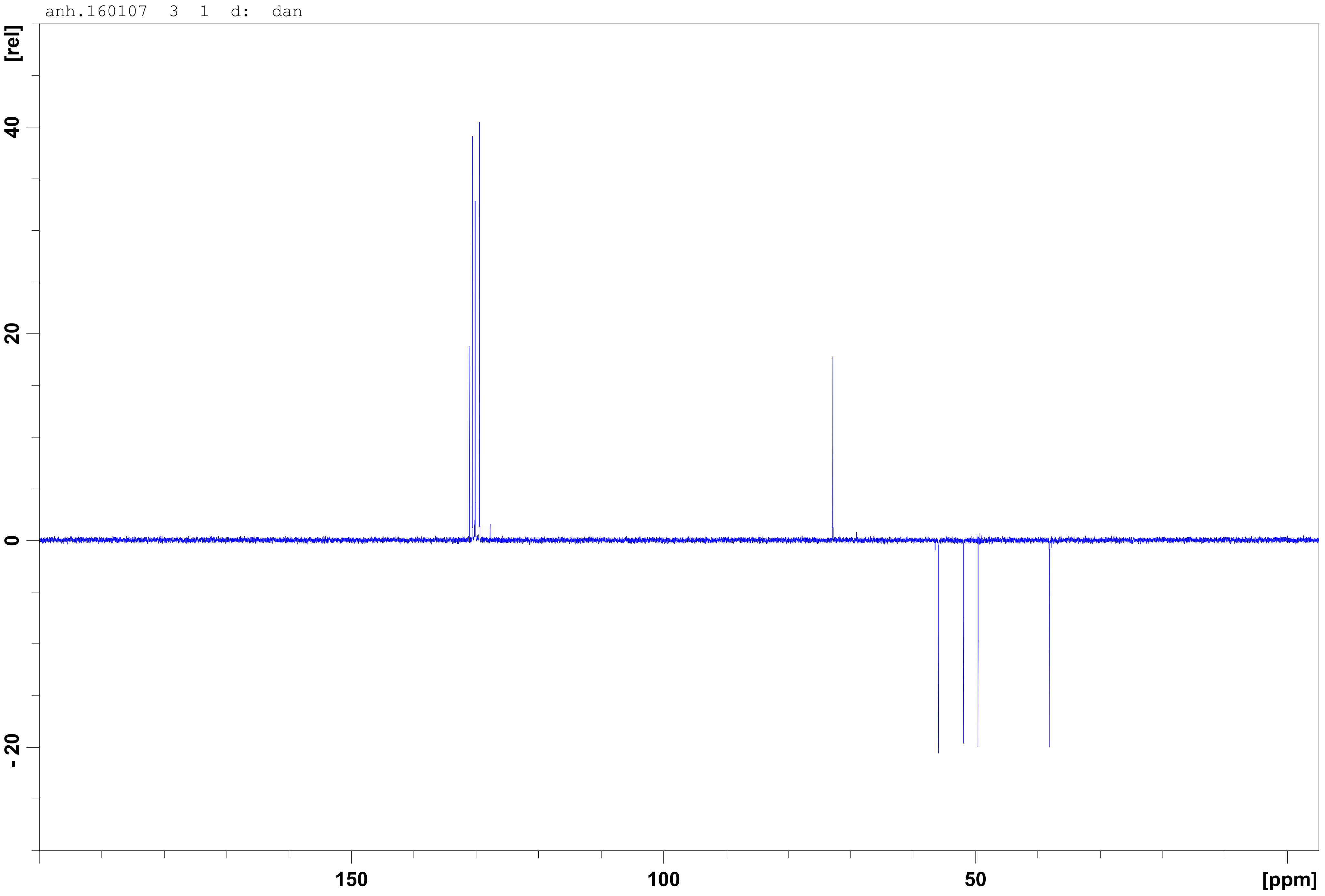

anh.160107 3 1 d: dan
[rel]
40
20
0
- 20
150
100
50
[ppm]

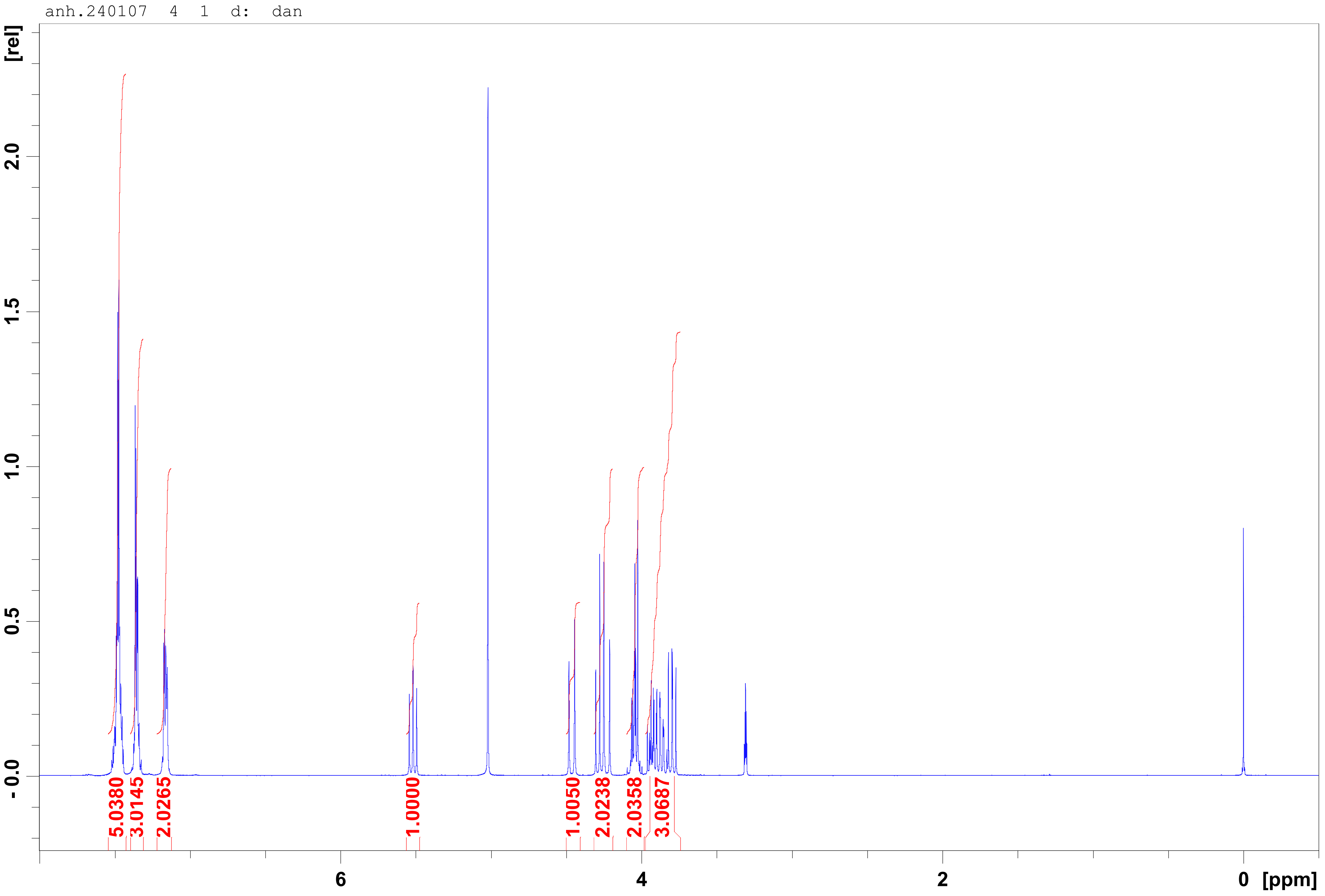
anh.240107 4 1 d: dan
[rel]
2.0
1.5
1.0
0.5
- 0.0
5.0380
3.0145
2.0265
1.0000
1.0050
2.0238
2.0358
3.0687
6
4
2
0 [ppm]

anh.240107 5 1 d: dan

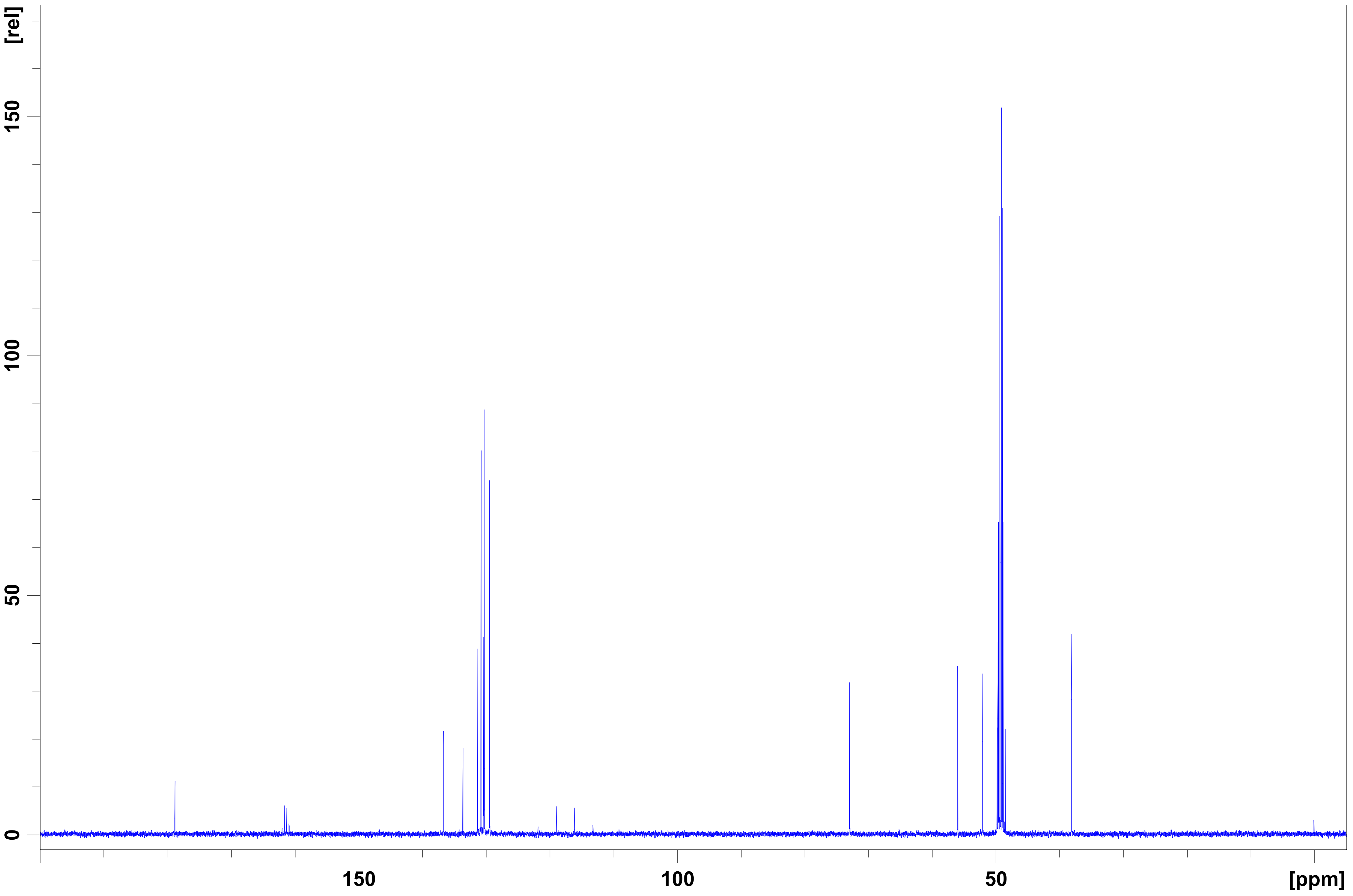

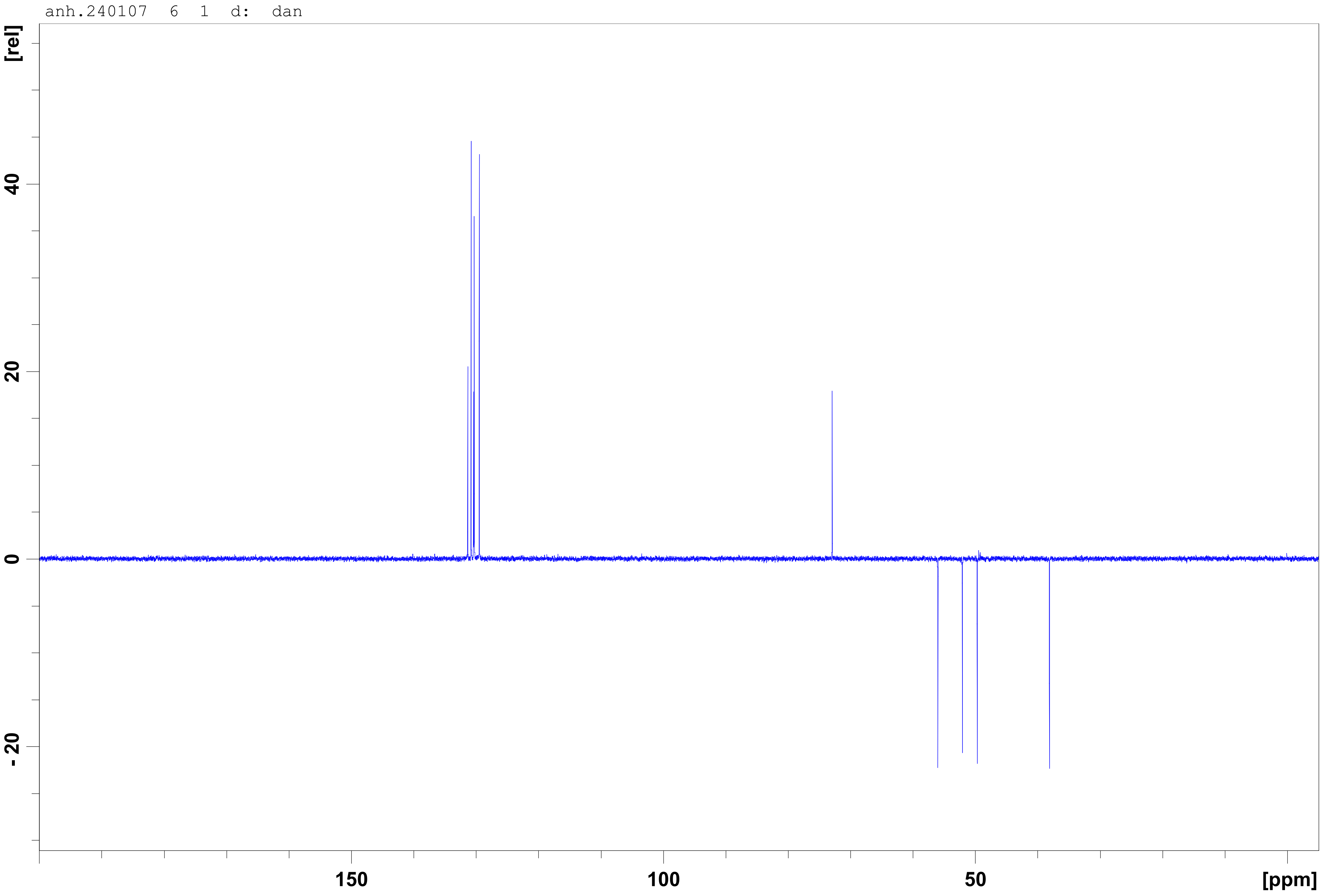
anh.240107 6 1 d: dan
[rel]
40
20
0
- 20
150
100
50
[ppm]

anh.240107 10 1 d: dan

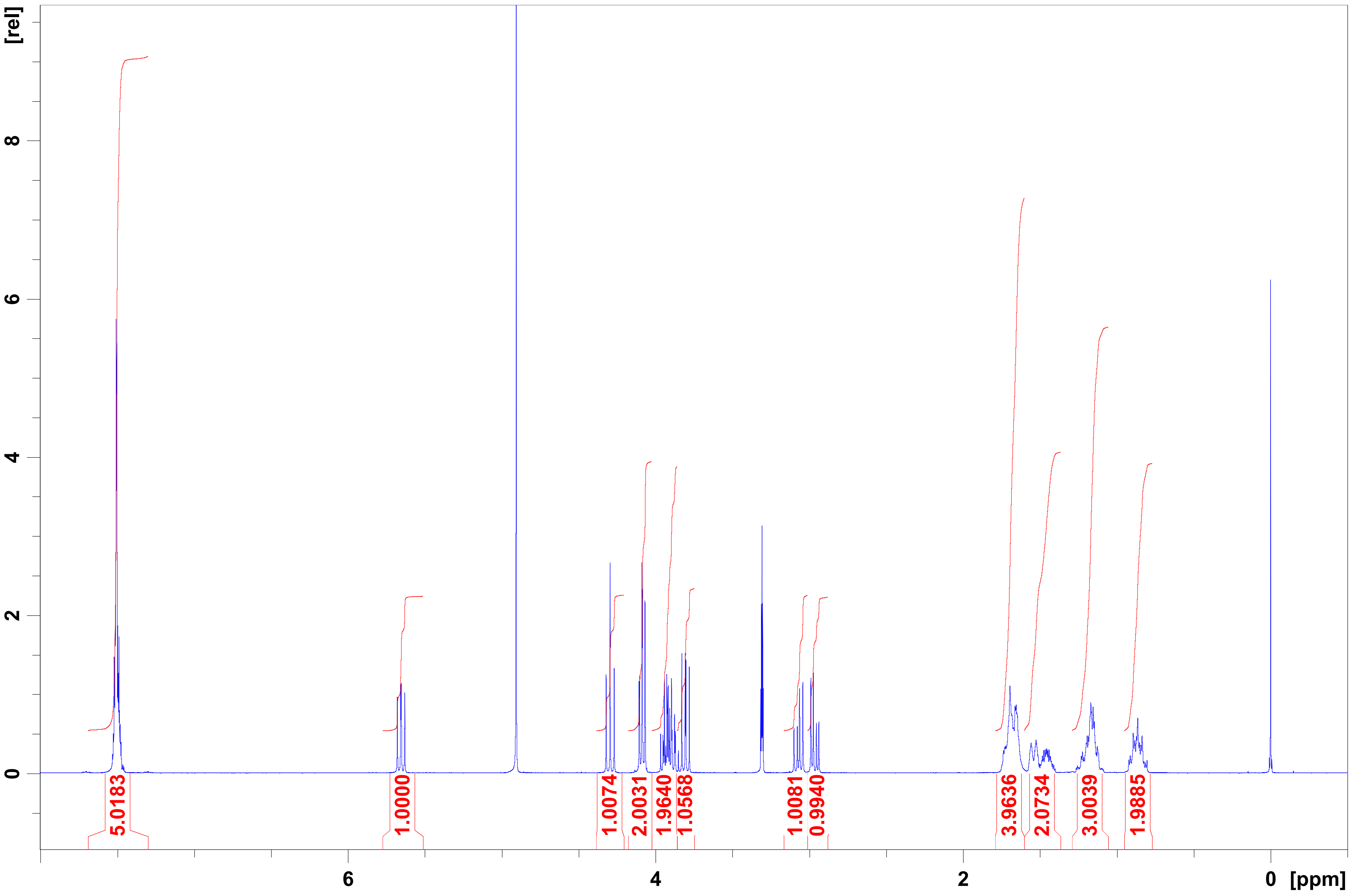

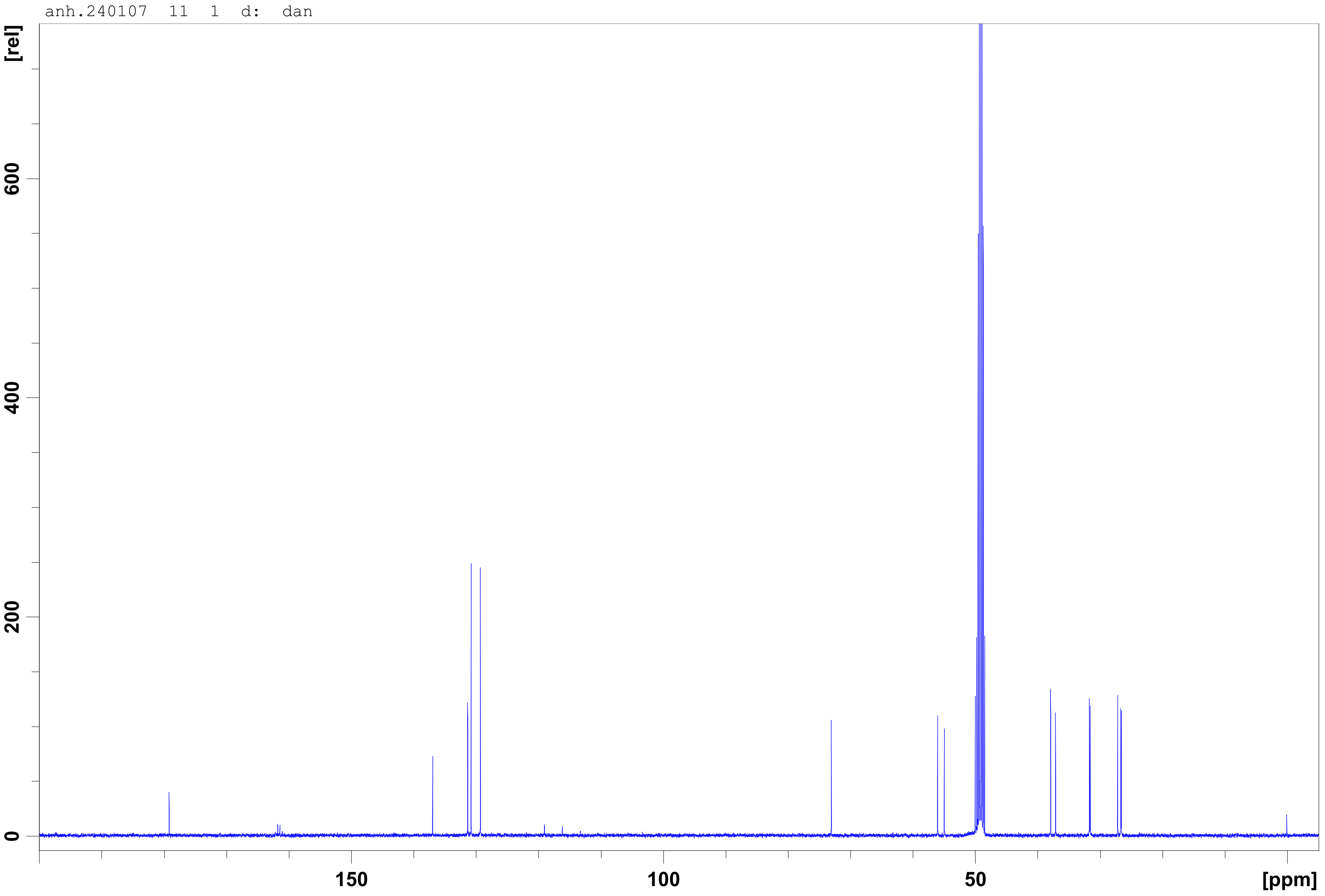
anh.240107 11 1 d: dan
[rel]
600
400
200
0
150
100
50
[ppm]

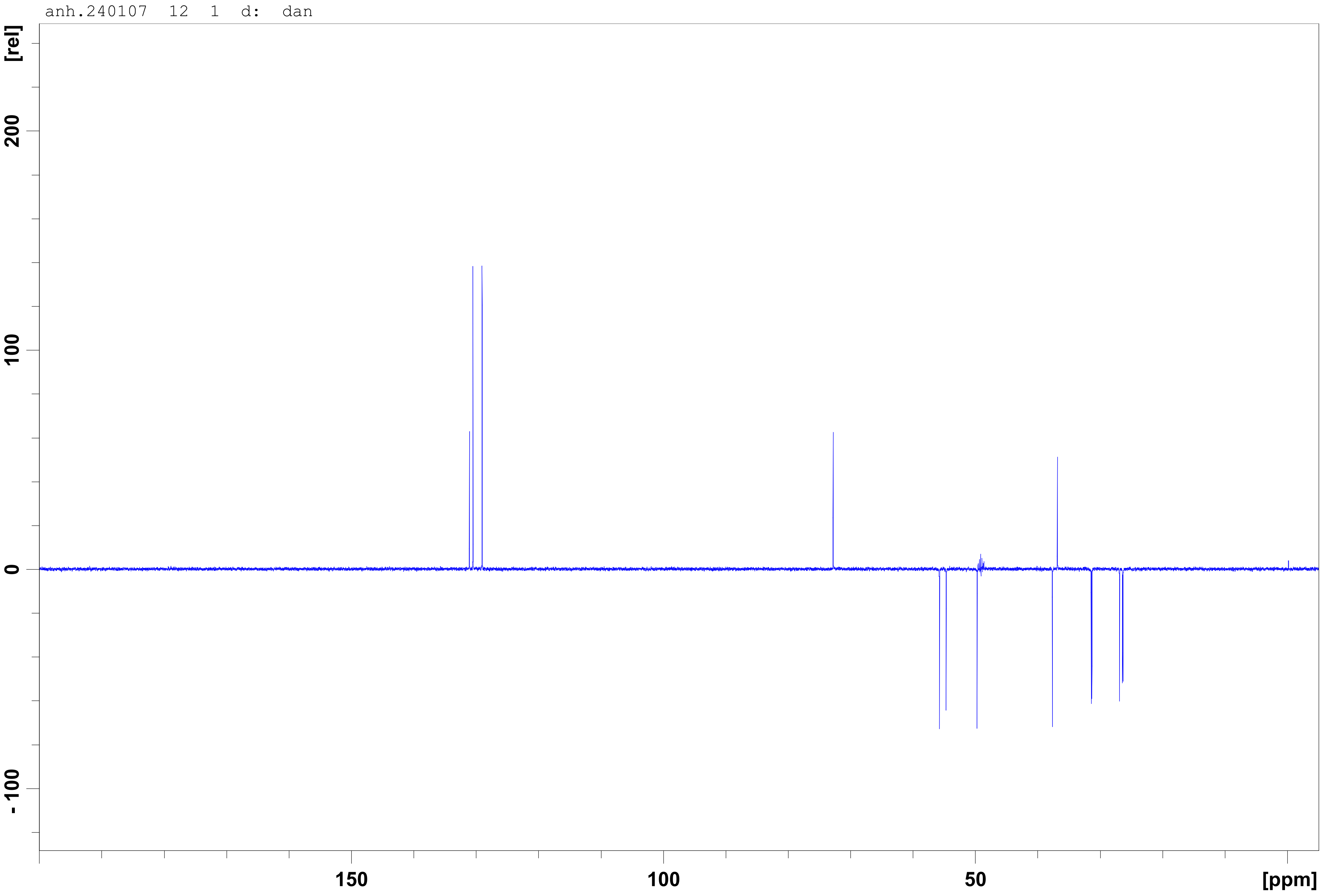

anh.240107 12 1 d: dan
[rel]
200
100
0
- 100
150
100
50
[ppm]

Table S1. Full assignment of the NMR spectral data obtained for compound 12.

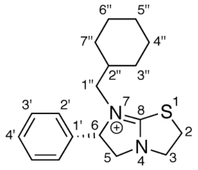

Position	$^{13}C^{a}$	$^{1}H^{a,b}$	COSY[a,c]	NOESY[a,c,d]	HSQC[a,c,e]	HMBC[a,c,f]
2	49.9	3.87 (m, A/B part of a larger spin system)	H-3A, H-3B	H-3A, H-3B	C-2	C-3, C-8
		3.93 (m, A/B part of a larger spin system)				
3	37.8	4.08 (m, A/B part of a larger spin system)	H-2	H-2	C-3	C-2, C-8
		4.10 (m, A/B part of a larger spin system)	H-2	H-2	C-3	
5	55.9	α: 3.80 (dd, $J_{5\alpha, 5\beta}$ =10.4, $J_{5\alpha, 6}$ =8.8)	H-5β, H-6	H-5β, H-2'	C-5	C-6, C-8, C-1'
		β: 4.29 (t, $J_{5\beta, 5\alpha}$ = 10.4, $J_{5\beta, 6}$ = 10.4)	H-5α, H-6	H-5α	C-5	C-6, C-8, C-1'
6	73.0	5.66 (dd, $J_{6, 5\beta}$ = 10.4, $J_{6; 5\alpha}$ = 8.8)	H-5α, H-5β, H-2', H-6'	H-2', H-5α, H-5β	C-6	C-5, C-2', C-6', C-1''
8	179.1					
1'	136.9					
2'	129.2	7.51 (m)	H-6	H-6, H-5α	C-2'	C-6
3'	130.7	7.49 (m)			C-3'	
4'	131.3	7.48 (m)			C-4'	
1''	54.9	α: 2.96 (dd, $J_{1''\alpha, 1''\beta}$ = 14.4, $J_{1''\alpha, 2''}$ = 6.29)	H-2'', H-1''β	1''β	C-1''	C-6, C-8, C-2'', C-7''
		β: 3.07 (dd, $J_{1''\beta, 1''\alpha}$ = 14.4, $J_{1''\beta, 2''}$ = 8.44)	H-2'', H-1''α	1''α	C-1''	
2''	37.0	1.46 (m)	H-1''α, H-1''β		C-2''	C-1''
3''	26.6	A: 1.55 (m)			C-3''	
		B: 1.15 (m)			C-3''	
4''	27.1	1.16 (m)	H-5''		C-4''	
5''	31.6	0.86 (m)	H-4''		C-5''	
6''	26.5	1.69 (m)			C-6''	
7''	31.5	A: 1.54 (m)			C-7''	C-1''
		B: 1.67 (m)			C-7''	

[a] ^{1}H (400 MHz) and ^{13}C (100 MHz) NMR spectral data obtained in CD_3OD. [b] Signal multiplicity and coupling constants in Hz are given in parentheses. [c] Signals correlate with ^{1}H resonance. [d] Mixing time 500 milliseconds. [e] Optimized for $^{1}J_{C,H}$ = 145 Hz. [f] Optimized for $^{n}J_{C,H}$ = 7.7 Hz.
